# Supplementary figures and images for: Increase in people’s behavioural risks for contracting COVID-19 during the 2021 New Year holiday season: longitudinal survey of the general population in Japan
Source: BMJ Open. 2022 Feb 3;12(2):e054770. doi: 10.1136/bmjopen-2021-054770 (PMC8814428; doi:10.1136/bmjopen-2021-054770)

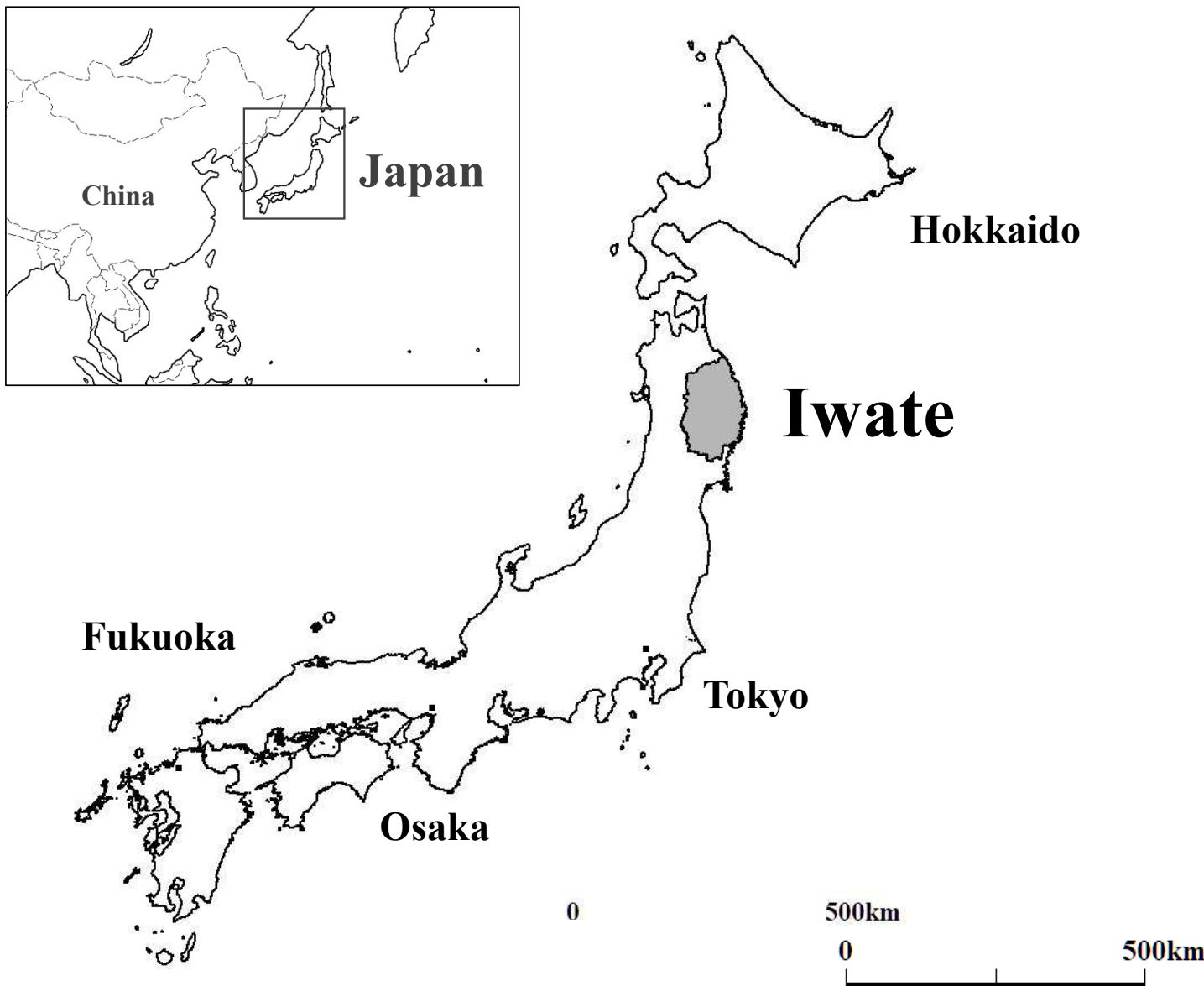

Supplement: Supplementary data [file bmjopen-2021-054770supp001.pdf]
