# Supplementary material for: Increase in people’s behavioural risks for contracting COVID-19 during the 2021 New Year holiday season: longitudinal survey of the general population in Japan
Source: BMJ Open. 2022 Feb 3;12(2):e054770. doi: 10.1136/bmjopen-2021-054770 (PMC8814428; doi:10.1136/bmjopen-2021-054770)
Supplement: Supplementary data [file bmjopen-2021-054770supp002.pdf]

Supplementary data

S1 Text. Detailed calculation of microCOVID.

The microCOVID project is a calculator to measure and numerically quantify the risk of getting COVID-19 from an activity or relationship in participants' daily life based on available evidence. 1 microCOVID means a one-in-a-million chance of getting COVID. Microcovids are computed by using three major factors: activity risk, person risk, and number of people. We obtained the value by multiplying person interactions: microCOVID = Activity Risk x Number of people x Person Risk for one person.

Activity risk indicates the chance that the activity will transmit COVID from one to the other person who currently has COVID. In accordance with a previous study conducted by The Behavioral Insights Team in the UK [1], we modified some items in the questionnaire by asking respondents “did you meet anyone outside your household (or support bubble) in a risky situation in the past week?”. A microCOVID value of zero was given to respondents who did not meet any person. Respondents who answered that they met someone went to the next questions asked about the details of the microCOVIDs value. Activity risk is a weighting based on coefficients how the interaction in the question is different from the reference value. Coefficients of risk are assigned to other types of interaction which differ from that reference value (if one person who has COVID, for 1 hour, indoors, unmasked, at 3 feet, there is a baseline 9% chance of transmission per hour) based on the following factors: duration of interaction, mask wearing (respondents and other persons), indoor/outdoor environment (indoor, outdoor, indoor with a HEPA filter, a train with air filtration, an airplane, a moving car with the windows rolled down, a space with one or more sides open to the outdoors), distance from each other, volume of conversation, and frequency (times a week). We calculated Activity Risk by multiplying 9%: Activity risk = coefficients x 9% (**S1 Table**).

BMJ Publishing Group Limited (BMJ) disclaims all liability and responsibility arising from any reliance placed on this supplemental material which has been supplied by the author(s)

With regard to the number of people, we asked respondents "how many people were there within a 5 meter radius of the scene?".

Person risk represents the chance that the other person currently has COVID based on overall prevalence in the person's area and recent behaviors of the person. Because the chance of a person having COVID depends not only on whether that person has any symptoms but also on the actions and choices of that person in the past 10 days, we used three different methods for estimating the probability of a person having COVID: a basic method, an intermediate method and an advanced method. The basic method is just an assumption that a person is "average" for their region. The probability of your friend having COVID is the probability of anyone in their geographic area having COVID. New infections in the past week were calculated as follows: new infections in the past week = reported cases x underreporting factor x delay factor. Based on that value, we estimated the person risk as follows: person risk (basic) = New infections in the past week / population in millions. The intermediate method is a quick adjustment for the risk a person being exposed at his/her workplace. The advance method involves adding up the risk of each individual activity that the person has done recently with consideration of three categories: socializing, errands, and work. In the intermediate and advanced methods, we calculated person risk by multiplying person risk (basic) by weighting coefficients in the following 12 items (**S1 Table**): "being an essential worker (working in health care, transportation or retailing)", "living alone and only going to grocery stores for shopping", "living in a closed pod of 4 people", "living in a closed pod of 10 people", "living in a closed pod of 20 people", "having gone to a bar in the past 10 days", "going to a bar frequently", "having been a patient with COVID-19", "you and other persons living with you only going to grocery stores for shopping", "other persons living with

Takahashi S, *et al. BMJ Open* 2022; 12:e054770. doi: 10.1136/bmjopen-2021-054770

you not being essential workers”, “not have any interaction with others and other persons living with you who are essential workers”, and “not applicable for those items”.

Takahashi S, *et al. BMJ Open* 2022; 12:e054770. doi: 10.1136/bmjopen-2021-054770

S1 Table. Weighting coefficient in items in each factor in activity and person risk.

Risk	Factors	Items	Coefficient
**Activity Risk**	**Respondents' mask**	No mask or inappropriate usage	1
		Polyester or polyurethane mask	0.5
		Cloth or bandanna	0.25
		Surgical mask, non-woven mask	0.25
		N95 mask	0.1
	**Other person's mask**	No mask or inappropriate usage	1
		Polyester or polyurethane mask	1
		Cloth or bandanna	1
		Surgical mask, non-woven mask	0.5
		N95 mask	0.1
	**Indoor/outdoor environment**	Indoor without ventilation	1
		Outdoor	0.05
		Indoor with a HEPA filter	0.25
		A train with air filtration	0.25
		An airplane	0.17
		A moving car with the windows rolled down	0.25
		A space with one or more sides open to the outdoors	0.25
	**Distance from each other**	< 0.3 meters apart	2

		About 1 meter apart	1
		2+ meters apart	0.5
		3+ meters apart	0.25
	**Volume of conversation**	Not talking	0.2
		Normal conversation	1
		Loud talking (shouting, talking over music, singing)	5
**Person Risk**	**Intermediate and Advanced Method**	Being an essential workers	2
		Living alone and only going to grocery stores for shopping	0.011
		Living in a closed pod of 4 people	0.021
		Living in a closed pod of 10 people	0.041
		Living in a closed pod of 20 people	0.075
		Having gone to a bar in the past 10 days	14.5
		Going to a bar frequently	0.9
		Having been a patient with COVID-19	1000000
		You and other persons living with you only going to grocery stores for shopping	0.014
		Other persons living with you not being essential workers	0.5
		Not have any interaction with others and other persons living with you who are essential workers	0.61
		Not applicable for those items	1

S2 Table. Results of analysis using models for risk trajectories by multiple imputation methods (n=9,876).

		Model for transition to high risk (n=8,113)		Model for high risk persistence (n=1,763)	
		**OR (95% CI)**	**P-value**	**OR (95% CI)**	**P-value**
**Age groups**	**Young (ref: elderly)**	1.96 (1.19 - 3.24)	0.008	1.59 (0.90 - 2.81)	0.111
	**Middle age**	1.51 (0.93 - 2.44)	0.092	1.49 (0.86 - 2.57)	0.157
**Sex**	**Women (ref: men)**	1.05 (0.80 - 1.37)	0.733	1.25 (0.91 - 1.71)	0.17
**Area**	**Inland areas (ref: coastal and mountainous areas)**	0.99 (0.75 - 1.32)	0.958	0.83 (0.62 - 1.11)	0.207
**Occupation**	**Health care workers (ref: government workers)**	2.66 (1.76 - 4.01)	<0.001	1.78 (1.17 - 2.73)	0.008
	**Service**	1.18 (0.78 - 1.78)	0.437	1.51 (0.97 - 2.35)	0.07
	**Schools**	1.17 (0.69 - 1.98)	0.569	2.62 (1.59 - 4.34)	<0.001
	**Others**	0.56 (0.35 - 0.88)	0.013	1.23 (0.72 - 2.10)	0.451
**Rate of decrease in eating out compared with that in the past year**	**Decreased by 50-70% (ref: decreased by 80% or more)**	1.22 (0.91 - 1.64)	0.183	1.03 (0.76 - 1.40)	0.83
	**Decreased by 40% or less**	1.02 (0.75 - 1.39)	0.888	1.21 (0.89 - 1.64)	0.22
**Homecoming in the New Year's holiday season**	**Yes (ref: no)**	1.04 (0.75 - 1.44)	0.809	1.17 (0.85 - 1.59)	0.334
**The first shrine visit of the year**	**Do not visit every year (ref: not visited on this year for prevention)**	1.08 (0.76 - 1.55)	0.664	1.81 (1.23 - 2.66)	0.003
	**Visited**	1.03 (0.78 - 1.35)	0.853	1.12 (0.85 - 1.49)	0.414
**Measures in the second state of emergency**	**Lower (ref: higher)**	1.59 (1.04 - 2.44)	0.032	1.28 (0.85 - 1.94)	0.244
	**Same**	0.90 (0.65 - 1.23)	0.487	0.77 (0.57 - 1.04)	0.085

Abbreviations: CI, confidence interval; OR, odds ratio

Takahashi S, *et al. BMJ Open* 2022; 12:e054770. doi: 10.1136/bmjopen-2021-054770

S3 Table. Comparison of characteristics of the participants in the first survey and the characteristics of the whole population in the 2020 local census in Iwate.

		2020 local census	All analytic sample in the first survey
		n (%)	n (%)
**Sex**	**Men**	585077 (48.3)	2692 (27.6)
	**Women**	627124 (51.7)	7049 (72.4)
**Age classes**	**Young**	396943 (32.7)	2765 (28.4)
	**Middle age**	315660 (26.0)	5802 (59.6)
	**Elderly**	492455 (40.6)	1174 (12.1)
	**Unknown**	7143 (0.6)	Not applicable
**Area**	**Inland areas**	928199 (76.6)	7859 (80.7)
	**Coastal and mountainous areas**	284002 (23.4)	1882 (19.3)

S4 Table. Results of analysis using models for risk trajectories with survey weights (n=9,741)

		Model for transition to high risk (n=8030)		Model for high risk persistence (n=1711)	
		**OR (95% CI)**	**P-value**	**OR (95% CI)**	**P-value**
**Age groups**	**Young (ref: elderly)**	2.56 (1.51 - 4.33)	<0.001	1.37 (0.71 - 2.65)	0.342
	**Middle age**	1.78 (0.99 - 3.19)	0.052	1.47 (0.72 - 2.98)	0.288
**Sex**	**Women (ref: men)**	1.09 (0.76 - 1.56)	0.652	1.31 (0.88 - 1.95)	0.19
**Area**	**Inland areas (ref: coastal and mountainous areas)**	1.75 (1.14 - 2.70)	0.011	1.00 (0.65 - 1.54)	0.987
**Occupation**	**Health care workers (ref: government workers)**	1.74 (1.01 - 2.98)	0.045	1.42 (0.75 - 2.66)	0.28
	**Service**	0.99 (0.60 - 1.63)	0.958	1.08 (0.57 - 2.08)	0.808
	**Schools**	0.53 (0.24 - 1.15)	0.107	1.60 (0.79 - 3.25)	0.19
	**Others**	0.65 (0.37 - 1.12)	0.121	0.54 (0.22 - 1.30)	0.168
**Rate of decrease in eating out compared with that in the past year**	**Decreased by 50-70% (ref: decreased by 80% or more)**	1.17 (0.77 - 1.78)	0.467	1.40 (0.88 - 2.23)	0.153
	**Decreased by 40% or less**	1.38 (0.92 - 2.07)	0.122	1.44 (0.89 - 2.34)	0.139
**Homecoming in the New Year's holiday season**	**Yes (ref: no)**	0.86 (0.56 - 1.33)	0.501	1.05 (0.67 - 1.63)	0.847
**The first shrine visit of the year**	**Do not visit every year (ref: not visited on this year for prevention)**	0.99 (0.59 - 1.67)	0.98	2.72 (1.48 - 5.01)	0.001
	**Visited**	1.03 (0.72 - 1.49)	0.868	2.05 (1.26 - 3.34)	0.004
**Measures in the second state of emergency**	**Lower (ref: higher)**	1.44 (0.85 - 2.44)	0.178	1.33 (0.73 - 2.43)	0.358
	**Same**	0.73 (0.48 - 1.09)	0.127	0.66 (0.41 - 1.05)	0.081

Abbreviations: CI, confidence interval; OR, odds ratio

Takahashi S, *et al. BMJ Open* 2022; 12:e054770. doi: 10.1136/bmjopen-2021-054770

S5 Table. Comparison of baseline characteristics of the non-participants and participants at the follow-up survey

		Missing	Non-participants (n=15055)	Study participants (n=10356)	*P* value
		n (%)	n (%)	n (%)	
**Age groups**	**Young**	0 (0.0)	4638 (30.8)	2950 (28.5)	<0.001
	**Middle age**		8544 (56.8)	6153 (59.4)	
	**Elderly**		1873 (12.4)	1253 (12.1)	
**Sex**	**Women**	117 (0.0)	10215 (68.2)	7420 (72.0)	<0.001
**Area**	**Inland areas**	0 (0.0)	12322 (81.8)	8311 (80.3)	<0.001
**Occupation**	**Health care workers**	0 (0.0)	3323 (22.1)	2117 (20.4)	<0.001
	**Service**		4992 (33.2)	3048 (29.4)	
	**Schools**		1158 (7.7)	859 (8.3)	
	**Others**		4202 (27.9)	3112 (30.1)	
	**Government workers**		1380 (9.2)	1220 (11.8)	

P values were calculated using the chi-squared test for categorical variables.

Takahashi S, *et al. BMJ Open* 2022; 12:e054770. doi: 10.1136/bmjopen-2021-054770

References

1. Yihan X, Mark E, Tania L, Lev T, Abigail M, Hugo H. A small number of people account for a large amount of coronavirus risk. In: team Tbi, ed. United Kingdom: The behavioural insights team, 2020.
