# Supplementary material for: Increase in people’s behavioural risks for contracting COVID-19 during the 2021 New Year holiday season: longitudinal survey of the general population in Japan
Source: BMJ Open. 2022 Feb 3;12(2):e054770. doi: 10.1136/bmjopen-2021-054770 (PMC8814428; doi:10.1136/bmjopen-2021-054770)
Supplement: Author's manuscript [file bmjopen-2021-054770.draft_revisions.pdf]

BMJ Open is committed to open peer review. As part of this commitment we make the peer review history of every article we publish publicly available.

When an article is published we post the peer reviewers' comments and the authors' responses online. We also post the versions of the paper that were used during peer review. These are the versions that the peer review comments apply to.

The versions of the paper that follow are the versions that were submitted during the peer review process. They are not the versions of record or the final published versions. They should not be cited or distributed as the published version of this manuscript.

BMJ Open is an open access journal and the full, final, typeset and author-corrected version of record of the manuscript is available on our site with no access controls, subscription charges or pay-per-view fees (http://bmjopen.bmj.com).

If you have any questions on BMJ Open's open peer review process please email info.bmjopen@bmj.com

BMJ Open

Increases in people's behavioral risks for contracting COVID-19 during the 2021 New Year holiday season in Japan

Journal:	*BMJ Open*
Manuscript ID	bmjopen-2021-054770
Article Type:	Original research
Date Submitted by the Author:	28-Jun-2021
Complete List of Authors:	Takahashi, Shuko; Iwate Prefecture Government, Department of Health and Welfare; Iwate Medical University<br>Yamada, Shohei; Iwate Prefecture Government, Department of Health and Welfare<br>Sasaki, Satoshi; Iwate Prefecture Government, Department of Health and Welfare<br>Minato, Yoichi; Iwate Prefecture Government, Department of Health and Welfare<br>Takahashi, Naomi; Iwate Medical University Faculty of Medicine Graduate School of Medicine, Department of Hygiene and Preventive Medicine<br>Kudo, Keiichiro; Iwate Prefecture Government, Department of Health and Welfare<br>Masaru, Nohara; Iwate Prefecture, Department of Health and Welfare<br>Kawachi, Ichiro; Harvard University T H Chan School of Public Health, Department of Society Human Development and
Keywords:	COVID-19, EPIDEMIOLOGY, Health policy < HEALTH SERVICES ADMINISTRATION & MANAGEMENT, Risk management < HEALTH SERVICES ADMINISTRATION & MANAGEMENT, Public health < INFECTIOUS DISEASES

SCHOLARONE™
Manuscripts

I, the Submitting Author has the right to grant and does grant on behalf of all authors of the Work (as defined in the below author licence), an exclusive licence and/or a non-exclusive licence for contributions from authors who are: i) UK Crown employees; ii) where BMJ has agreed a CC-BY licence shall apply, and/or iii) in accordance with the terms applicable for US Federal Government officers or employees acting as part of their official duties; on a worldwide, perpetual, irrevocable, royalty-free basis to BMJ Publishing Group Ltd ("BMJ") its licensees and where the relevant Journal is co-owned by BMJ to the co-owners of the Journal, to publish the Work in this journal and any other BMJ products and to exploit all rights, as set out in our licence.

The Submitting Author accepts and understands that any supply made under these terms is made by BMJ to the Submitting Author unless you are acting as an employee on behalf of your employer or a postgraduate student of an affiliated institution which is paying any applicable article publishing charge ("APC") for Open Access articles. Where the Submitting Author wishes to make the Work available on an Open Access basis (and intends to pay the relevant APC), the terms of reuse of such Open Access shall be governed by a Creative Commons licence – details of these licences and which Creative Commons licence will apply to this Work are set out in our licence referred to above.

Other than as permitted in any relevant BMJ Author's Self Archiving Policies, I confirm this Work has not been accepted for publication elsewhere, is not being considered for publication elsewhere and does not duplicate material already published. I confirm all authors consent to publication of this Work and authorise the granting of this licence.

For peer review only

Increases in people's behavioral risks for contracting COVID-19 during the 2021 New Year holiday season in Japan

Shuko Takahashi [a, b] *, Shohei Yamada [a], Satoshi Sasaki [a], Yoichi Minato [a], Naomi Takahashi [c], Keiichiro Kudo [a], Masaru Nohara [a], and Ichiro Kawachi [d]

[a] Office of Medical Policy, Department of Health and Welfare, Iwate Prefecture Government, 10-1 Uchimaru, Morioka, Iwate 020-8570, Japan

[b] Division of Medical Education, Iwate Medical University, 1-1-1, Idaidori, Yahaba-cho, Shiwa-gun, Iwate 028-3694, Japan

[c] Graduate School of Medicine, Faculty of Medicine, Iwate Medical University, School of Medicine, 1-1-1, Idaidori, Yahaba-cho, Shiwa-gun, Iwate 028-3694, Japan

[d] Harvard. T.H. Chan School of Public Health, 401 Park Drive, Boston MA 02215, USA

* Corresponding author. Shuko Takahashi, MD, PhD. Office of Medical Policy, Department of Health and Welfare, Iwate Prefecture Government, 10-1 Uchimaru, Morioka, Iwate 020-8570, Japan.

Tel: +81-19-651-3111, Fax: +81-19-651-3111, E-mail: shutakahashi-iwt@umin.ac.jp

Keywords; COVID-19, Epidemiology, Health policy, Risk management, Public health

For peer review only - http://bmjopen.bmj.com/site/about/guidelines.xhtml

Abstract

Objectives: There has been no study in Japan on the predictors of risk for acquiring SARS-CoV-2 infection based on people's behavior during the COVID-19 pandemic. The aim of this study was to document changes in risk behavior during the New Year's holiday season in 2021 and to identify factors associated with high-risk behavior for infection using a quantitative assessment tool.

Design: A longitudinal survey.

Setting: Multiphasic health check-ups for the general population in Iwate Prefecture.

Participants: Serial cross-sectional data were obtained using rapid online surveys of residents in Iwate Prefecture from December 4 to 7 in 2020 (baseline survey) and from February 5 to 7 in 2021 (followup survey). The data in those two surveys was available for a total of 9,741 participants.

Main outcome measures: We estimated each individual's risk of acquiring SARS-CoV-2 infection based on the microCOVID calculator. We defined four trajectories of individual risk behaviors based on the probabilities of remaining at low risk, increasing to high risk, improving to low risk, and persistence of high risk.

Results: Among people in the low risk group in the first survey, 3.6% increased to high risk, while high risk persisted in 80.0% of people who were in the high risk group at baseline. While health care workers were significantly more likely to be represented in both the increasing risk and persistently high risk group, workers in the education setting were also associated with persistence of high risk (OR [95% confidence interval], 2.58 [1.52–4.39]; P <0.001).

For peer review only

CONCLUSIONS: In determining counter-measures against COVID-19 (as well as future outbreaks), health officials should take into account population changes in behavior during large-scale public events.

Strengths and limitations of this study

1. The large sample of participants who were followed at two different time points and the determination of predictors of risky behavior for COVID-19 during the New Year holiday season.
2. Serial cross-sectional data were obtained using rapid online surveys of residents.
3. The risk for infection increased in a small group (3.6%) of people during the holiday, while remaining persistently elevated in 80.0% of people.
4. Health care workers, teachers & students in schools, were groups at particularly high risk.
5. It was not determined questions correlated with healthy behaviors such as educational attainment or household income.

For peer review only
For peer review only - http://bmjopen.bmj.com/site/about/guidelines.xhtml

Introduction

Cases of COVID-19 spread worldwide soon after the first case was identified in China in 2019. The worldwide death toll from COVID-19 as of March 29, 2021 is 2,792,706 [1]. The first case of COVID-19 in Japan was confirmed on January 16, 2020, and cases then spread from urban areas to rural areas in Japan.

In December 2020, Japan experienced a third wave of outbreak which was much larger than the second wave, peaking at 8,045 new cases on January 8, 2021 [2]. The Japanese government declared a second state of emergency for Tokyo and its surrounding prefectures on January 7, 2021, and several measures were implemented including shortening the opening hours for bars and restaurants and encouraging tele-work to decrease the number of commuters by 70% [3].

One of the reasons for the increase in the number of COVID-19 cases in Japan was the movement of people during the traditional New Year holiday. New Year's Day is the most important holiday in Japan, when many people return to their hometowns to spend time with their family, relatives or local friends. Although there is a decrease in the movement of people because schools, businesses and workplaces are closed for several days, large crowds of people gather to pray at local shrines or temples on New Year's Day (called "Hatsumode" in Japanese) [4].

The Japanese government opted to not restrict the movement of people during the period before and after New Year's Day. Some people also ignored the government's request to refrain from unnecessary outings, while other people continued to take preventive measures against infection. Studies have shown that there are variations in the tendency to adopt protective measures such as social distancing based upon on people's age, gender, socioeconomic characteristics (educational attainment, occupation), and political ideology [5-

For peer review only

For peer review only - http://bmjopen.bmj.com/site/about/guidelines.xhtml

8]. In order to establish effective interventions during large-scale public events, there is a need to identify high risk groups.

The aim of our study was to determine different groups of individuals based on their trajectories of risk behavior during the New Year holiday season and to determine factors associated with behavior with a high risk for COVID-19 using a quantitative risk assessment tool, "microCOVID".

Materials and methods

Study area

Iwate Prefecture is located in the northeastern part of Japan (about 500 km from Tokyo) with a population of about 1.2 million (**S1 Fig**). The total number of COVID-19 cases as of March 30, 2021 was 614 with 30 cases of COVID-19-related deaths reported [9].

Data

Rapid online surveys of residents in Iwate Prefecture were conducted by the Office of Medical Policy in the Department of Health and Welfare in Iwate Prefecture using a popular social network platform called LINE (LINE Corporation, Tokyo, Japan). By using the LINE app, Iwate Prefecture has been providing information about COVID-19 every day since the beginning of the pandemic, including information on the number of new cases and characteristics of patients. A series of cross-sectional surveys were started in December 2020 to investigate people's behavior in order to prevent infection and promote health consciousness. The surveys have been conducted every two month. An online questionnaire was administered to a total of 100,958 people who had registered by the time of the baseline survey. We sent out

For peer review only - http://bmjopen.bmj.com/site/about/guidelines.xhtml

notification about the survey via the LINE platform on December 4, 2020, and 25,411 individuals answered from December 4 to 7 (participants rate, 25.2%). We conducted a second survey of registered people from February 5 to 7, 2021. We analyzed the results of these two surveys. After excluding individuals who were lost to follow-up in the second survey (14,998), those who were missing data needed for the calculation of microCOVID scores either in first or second survey (n=436), those who live outside Iwate (n=34) and those who had other missing values for analyses (n=135), data were available for a total of 9,741 participants (**Fig. 1**).

Ethics approval statement

The surveys were conducted in accordance with applicable Japanese law and Iwate Prefectural Government policy. Since this study was a secondary analysis using anonymized data from Iwate Prefecture, the study was exempt from IRB review.

Outcome

We applied a weighting system to calculate the level of behavioral risk for acquiring COVID-19 infection, called microCOVID [10]. MicroCOVID is a calculator to numerically quantify the risk of getting COVID-19 from daily activities. 1.0 microCOVID is equivalent to a one-in-a-million chance of getting COVID. Microcovids are computed by using three major factors: activity risk, person risk, and number of people with whom an individual interacts with (**S1 Text**). We obtained the microCOVID value for each person whereby the score = activity risk x number of people x person risk.

Activity risk indicates the chance that an activity will transmit COVID from a person who currently is infected with COVID. According to microCOVID, the transmission risk is

For peer review only

estimated to be “about 9% per hour” from spending more than 10 minutes indoors or in close proximity with an unmasked person who is COVID-positive. Coefficients of risk are assigned to other types of interaction which differ from that reference value. The coefficients were calculated based on the following factors: duration of interaction, mask wearing (respondents and other persons), indoor/outdoor environment, distance from each other, volume of conversation, and frequency (times a week) (**S1 Table**). With regard to the number of people, we asked respondents “how many people were there within a 5 meter radius of the scene?”. Person risk represents the probability that a random person is currently infected with COVID based on the overall prevalence in the person’s area as well as the recent behaviors of the person (**S1 Table**). We classified each individual’s risk level into low-risk versus high-risk for acquiring infection (low-risk group, ≤20 microCOVIDs; high-risk group, >20 microCOVIDs).

Covariates

Age, sex, municipality, occupation, frequency of eating out compared with the past year, returning home during the New Year holiday, visits to the local shrine/temple were included as independent variables in the analysis. Participants were divided into three age categories: young (people less than 39 years of age), middle age (people of 40 to 59 years of age) and elderly (people aged 60 years or older). Residential areas were classified into inland versus coastal/mountainous based on the geography of Iwate prefecture. Occupations were assessed by asking “what is your current job?”. Participants were divided into five groups: health care workers, workers in service industries (e.g., transportation, customer-facing occupations in the retail/hospitality sector, office workers), education sector (teachers or students), government workers, and all others (workers in manufacturing, farmers/agricultural workers,

For peer review only - http://bmjopen.bmj.com/site/about/guidelines.xhtml

workers in other jobs, or unemployed). The frequency of eating out compared with the past year was classified into three groups: "decreased by 80% or more", "decreased by 50-70%" and “decreased by 40% or less”. With regard to the first shrine visit of the year, participants were divided into three categories: “never observe the ceremony”, “visited” and “did not visit this year as a preventive measure”. We asked respondents about the extent of preventive measures adopted during the second state of emergency compared with that in the first one. The participants were divided into three groups: lower (lower degree or no preventive measure), same (same as the first one) and higher (higher degree).

Statistical analyses

For analysis of the trajectories of behavior risks, we calculated the risk scores for each individual in the first survey and that in the second survey. Baseline characteristics were compared for the low-risk and high-risk microCOVID groups in the second survey using the chi-squared test. Logistic regression analyses was conducted to identify the characteristics related to high-risk behavior. Separate models were used to determine the predictors of each type of trajectory. We imputed missing covariate data by multiple imputation using Markov Chain Monte Carlo method, creating five imputed datasets. We used the Statistical Package for Social Sciences (SPSS) software program version 25.0 (IBM, Chicago, IL, USA) for all analyses. All statistical tests described were two-sided, and analysis items with P-values <0.05 were considered statistically significant.

For peer review only - http://bmjopen.bmj.com/site/about/guidelines.xhtml

Patient and public involvement

No funding was available for patient or public involvement in this project. No patients were involved in setting the research question or the outcome measures. Patients were not invited in the design, or conduct, or reporting, or dissemination plans of our research.

Results

Fig. 2 shows trajectories of behavioral risks from the first survey to the second survey. In December 2020, 17.6% of the respondents were classified into the high-risk behavior category. Two months after the first survey (straddling the New Year holidays), 3.6% of the low-risk individuals in the initial survey had transitioned to high risk, while 80.0% of the individuals in the high-risk behavior category in the baseline survey remained in the high-risk group.

Table 1 shows the baseline characteristics of participants in the low-risk group and high-risk group at the baseline survey. Individuals in the low-risk group at the baseline survey who later transitioned to high risk included a higher proportion of young people compared to individuals who remained in the low-risk group. Health care workers were also over-represented in the group who transitioned to high risk. The high risk group at baseline also included people in education (teachers and students), as well as individuals who reported never observing traditional visits to shrines/temples during New Year.

For peer review only

Table 1. Baseline characteristics of participants in the first survey (n=9,741).

		Low risk in the first survey (n=8030)				High risk in the first survey (n=1711)			
		Remained in low risk (n=7744)	Increased to high risk (n=286)			Improved to low risk (n=1368)	Persistent high risk (n=343)		
		n (%)	n (%)	*P* value		n (%)	n (%)	*P* value	
Age groups	Young	2127 (27.5)	108 (37.8)	<0.001	*	414 (30.3)	116 (33.8)	0.085	
	Middle age	4577 (59.1)	161 (56.3)			852 (62.3)	212 (61.8)		
	Elderly	1040 (13.4)	17 (5.9)			102 (7.5)	15 (4.4)		
Sex	Women	5476 (70.7)	211 (73.8)	0.263		1078 (78.8)	284 (82.8)	0.100	
Area	Inland areas	6250 (80.7)	232 (81.1)	0.863		1107 (80.9)	270 (78.7)	0.357	
Occupation	Health care workers	1274 (16.5)	100 (35.0)	<0.001	*	519 (37.9)	146 (42.6)	0.001	*
	Service	2295 (29.6)	87 (30.4)			385 (28.1)	91 (26.5)		
	Schools	600 (7.7)	24 (8.4)			116 (8.5)	48 (14.0)		
	Others	2671 (34.5)	45 (15.7)			167 (12.2)	30 (8.7)		
	Government workers	904 (11.7)	30 (10.5)			181 (13.2)	28 (8.2)		
Rate of decrease in eating out compared with that in the past year	Decreased by 80% or more	4517 (58.3)	159 (55.6)	0.378		781 (57.1)	1 (34.3)	0.343	
	Decreased by 50-70%	1673 (21.6)	60 (21.0)			296 (21.6)	86 (25.1)		
	Decreased by 40% or less	1554 (20.1)	67 (23.4)			291 (21.3)	74 (21.6)		
Homecoming in the New Year's holiday season	Yes	1189 (15.4)	50 (17.5)	0.328		231 (16.9)	70 (20.4)	0.126	
The first shrine visit of the year	Do not visit every year	1135 (14.7)	43 (15.0)	0.954		172 (12.6)	70 (20.4)	0.001	*
	Visited	3809 (49.2)	142 (49.7)			734 (53.7)	174 (50.7)		
	Did not visit this year for prevention	2800 (36.2)	101 (35.3)			462 (33.8)	99 (28.9)		
Measures in the second state of emergency	Lower	741 (9.6)	43 (15.0)	0.007	*	155 (11.3)	59 (17.2)	0.003	*
	Same	5345 (69.0)	180 (62.9)			933 (68.2)	204 (59.5)		

For peer review only

For peer review only - http://bmjopen.bmj.com/site/about/guidelines.xhtml

**Higher**	1658 (21.4)	63 (22.0)		280 (20.5)	80 (23.3)

Categorical variables are presented as number of cases (%).

P values were calculated using the chi-squared test for categorical variables.

[*] Statistically significant differences between two groups.

For peer review only - http://bmjopen.bmj.com/site/about/guidelines.xhtml

Table 2 summarizes the predictors of the trajectories of behavioral risks in the two models. With regard to transitioning to high risk, younger individuals as well as individuals with practicing a lower degree of preventive measures during the second state of emergency had significantly higher odds ratios (ORs). With regard to occupation, both health care workers and people in educational settings were more likely to be in the group with persistent high risk (OR [95% confidence interval (CI)], 2.58 [1.52–4.39]; P <0.001). Results were similar in the analyses with multiple imputation for missing data (n= 9,876) (**S2 Table**).

For peer review only

For peer review only - http://bmjopen.bmj.com/site/about/guidelines.xhtml

Table 2. Results of analysis using models for risk trajectories (n=9,741)

		Model for transition to high risk (n=8030)		Model for high risk persistence (n=1711)	
		**OR (95% CI)**	**P-value**	**OR (95% CI)**	**P-value**
**Age groups**	**Young (ref: elderly)**	2.22 (1.29 - 3.79)	0.004	1.54 (0.84 - 2.80)	0.16
	**Middle age**	1.60 (0.96 - 2.69)	0.073	1.50 (0.84 - 2.68)	0.166
**Sex**	**Women (ref: men)**	1.01 (0.76 - 1.33)	0.952	1.26 (0.91 - 1.74)	0.169
**Area**	**Inland areas (ref: coastal and mountainous areas)**	1.07 (0.79 - 1.46)	0.662	0.86 (0.63 - 1.16)	0.309
**Occupation**	**Health care workers (ref: government workers)**	2.49 (1.62 - 3.82)	<0.001	1.85 (1.18 - 2.90)	0.007
	**Service**	1.16 (0.76 - 1.78)	0.502	1.55 (0.97 - 2.47)	0.067
	**Schools**	1.11 (0.64 - 1.93)	0.709	2.58 (1.52 - 4.39)	<0.001
	**Others**	0.55 (0.34 - 0.89)	0.016	1.20 (0.68 - 2.12)	0.525
**Rate of decrease in eating out compared with that in the past year**	**Decreased by 50-70% (ref: decreased by 80% or more)**	0.98 (0.72 - 1.34)	0.918	1.24 (0.92 - 1.68)	0.158
	**Decreased by 40% or less**	1.25 (0.92 - 1.68)	0.151	1.04 (0.76 - 1.43)	0.8
**Homecoming in the New Year's holiday season**	**Yes (ref: no)**	1.06 (0.77 - 1.46)	0.736	1.18 (0.86 - 1.61)	0.299
**The first shrine visit of the year**	**Do not visit every year (ref: not visited on this year for prevention)**	1.05 (0.73 - 1.51)	0.805	1.83 (1.28 - 2.61)	0.001
	**Visited**	1.02 (0.79 - 1.33)	0.871	1.09 (0.83 - 1.44)	0.536
**Measures in the second state of emergency**	**Lower (ref: higher)**	1.62 (1.08 - 2.43)	0.021	1.28 (0.85 - 1.91)	0.233
	**Same**	0.92 (0.69 - 1.24)	0.592	0.75 (0.56 - 1.01)	0.056

Abbreviations: CI, confidence interval; OR, odds ratio

For peer review only

For peer review only - http://bmjopen.bmj.com/site/about/guidelines.xhtml

Discussion

We evaluated the predictors of high-risk behavior for SARS-CoV-2 infection during the Japanese New Year's holiday season in one prefecture. Overall, only a small percentage (<4%) of individuals transitioned from low risk (before the holidays) to high risk. However, the majority of individuals (80%) who were in the high risk behavior group at baseline remained high risk at follow-up.

The characteristics of individuals transitioning to high-risk behavior included younger age, people working in the health care sector, as well as individuals who admitted to practicing a lower degree of preventive measures during the state of emergency. Risk factors for persistently high risk were mainly based on occupation, viz. working in the health care sector and education sector.

Factors related to preventive knowledge and behavior for COVID-19 have been examined in several studies [5-8]. Shahnazi et al. examined the associations between preventive behaviors for COVID-19 and health belief factors, fatalistic beliefs and demographic factors among 750 Iranian adults during the period from March 11 to 16 in 2020. They showed that the adoption of preventive behavior was higher in women than men and higher in urban dwellers than rural dwellers [5]. Chen et al examined the differences between preventive behaviors for COVID-19 of urban and rural residents in China (n=1591) from January 31 to February 4 in 2020 [6]. Their results showed that rural residents were less likely to practice preventive behaviors. Li et al. examined the associations of Internet use, risk awareness, and demographic characteristics with engagement in preventative behaviors in the U.S. (n=979) from April 10 to April 14 in 2020 [7]. They showed that woman and older participants were more likely to adopt preventive behavior, and conversely more educated participants were less likely to adopt preventive behavior. An et al. investigated

For peer review only - http://bmjopen.bmj.com/site/about/guidelines.xhtml

characteristics related to positive and negative attitudes towards social distancing as a preventive measure in the U.S. (n=1074) in May 2020 [8]. They showed that female sex, older ages (65 years or older) and Democratic political party support were associated with a more positive attitude toward social distancing. Our study adds to the literature by tracking changes in population behavior during a national public holiday.

Identifying high risk occupational settings for acquiring COVID-19 infection remains an important goal for policy, e.g., prioritizing vaccinations. Health care work was recognized as a high-risk occupation during the 2009 influenza A (H1N1) pandemic [11]. Occupational risk in the current COVID-19 pandemic have been examined in several studies [12-14]. Nguyen et al. showed that the hazard ratio of the risk for COVID-19 was 3.40 [3.37-3.43] among front-line health-care workers [13]. Mutambudzi et al. reported that the risk ratio for testing positive for COVID-19 or death caused by COVID-19 was 7.43 [5.52-10.0] among healthcare workers [14]. These results are consistent with our results showing that health care workers were over-represented in both the persistently high risk group as well as the group transitioning from low to high risk. Although we could not conduct a study to validate the assessment tool due to an insufficient number of COVID-19 patients (n=15) among the respondents, an ecological survey by occupation that was conducted after the baseline survey showed that the proportion of COVID-19 infections among health care workers was high in Iwate prefecture.

Interestingly, people in the education sector had substantially higher ORs for high risk persistence even though they did not gather in schools for three weeks during the winter vacation. After our investigation period, which included 2 weeks after the second survey during the period from December 8, 2020 to February 21, 2021, about 14% of the total

For peer review only - http://bmjopen.bmj.com/site/about/guidelines.xhtml

number of COVID19 patients was reported to be among teachers and school-age children in Iwate. A meta-analysis showed that children and adolescents have lower susceptibility to SARS-CoV-2; the pooled odds ratio of being an infected contact in children compared with that in adults was 0.56 [0.37-0.85] [15]. A study on contact-tracing showed a lower level of transmission from index cases of children or teachers [16]. In those studies, the susceptibility to and transmission of SARS-CoV-2 in children or adolescents were compared to those in adults, and there was no comparison of the risks for teachers and students with the risks for other workers. Nguyen et al. also reported that social and education workers had a significantly higher risk for COVID-19 than did non-essential workers in the U.K and the U.S. [13]. The government in Japan closed schools at the start of the first wave of COVID-19 but did not request schools to close in the second and third waves, possibly based on the prediction of a low rate of infections in schools and less severe cases of COVID-19 in children [17]. Although fewer cases of infection and death have been reported in children and adolescents [18], a study conducted in Sweden showed that parents, teachers and partners exposed to open schools had significantly higher ORs than those in closed schools [19].

Initially, we hypothesized that people who made visits to shrines during the holidays (i.e., mingling with crowds) would have high risk for SARS-CoV-2 infection. In fact, our study did not suggest increased risk, possibly because of near universal mask-wearing, and the fact that praying at shrines & temples occurs outdoors. On the other hand, although there are some other risky behaviors that typically occur during the New Year holiday season in Japan, we could not collect detailed information on these events, e.g., “Bonen-kai” (end of year parties in workplaces) [20], and large indoor gatherings of extended families during the New Year.

For peer review only - http://bmjopen.bmj.com/site/about/guidelines.xhtml

Limitations

The present study had several limitations. First, we did not ask questions correlated with healthy behaviors such as educational attainment or household income. We also did not ask questions about job titles or occupational classes, which might influence risk of exposure to potentially high-risk environments. Second, there may have been selection bias since the respondents were limited to people registered in a health information program about COVID-19 maintained by the Iwate Prefectural government. We compared characteristics of the participants at baseline (December 2020) with characteristics of the whole population in Iwate in 2020 (**S3 Table**). While the percentages of young adults and elderly were smaller in our study than in the census data, the percentages of middle-aged people and women were higher in our study than in the census data (**S3 Table**). There was a large percentage of older women in the participants in our study. Despite these limitations, the important strengths of our study were the large sample of participants who were followed at two different time points and the determination of predictors of risky behavior for COVID-19 during the New Year holiday season. This enabled us to determine trajectories of high-risk behaviors straddling a large-scale public event.

Conclusion

Health care workers and people in the education sector (teachers and pupils) were found to have the highest behavioral risk during the New Year holiday season in Japan. Our results offer some clues for who should be prioritized for COVID19 vaccination as the program is rolled out in Japan.

For peer review only

Acknowledgments

The authors would like to thank the members in the Office of Medical Policy in the Department of Health and Welfare as well as the health care workers who contributed to active epidemiological investigation. The work was also supported by JSPS KAKENHI Grant Numbers JP20K18858. The funders had no role in the study design, data collection and analysis, decision to publish, or preparation of the manuscript.

Contributors

The authors indicate

Funding

The work was also supported by JSPS KAKENHI Grant Numbers JP20K18858. The funders had no role in the study design, data collection and analysis, decision to publish, or preparation of the manuscript.

Competing Interests

The authors indicated no potential conflicts of interest.

Ethical approval

The surveys were conducted in accordance with applicable Japanese law and Iwate Prefectural Government policy. Since this study was a secondary analysis using anonymized data from Iwate Prefecture, the study was exempt from IRB review.

For peer review only

For peer review only - http://bmjopen.bmj.com/site/about/guidelines.xhtml

Data sharing

The data that support the findings of this study are available from corresponding author (Dr. Shuko Takahashi) and Division of the Health and National Health Insurance in the Department of Health and Welfare, Iwate Prefecture Government but restrictions apply to the availability of these data, which were used under license for the current study, and so are not publicly available. Data are however available from the authors upon reasonable request and with permission of Division of the National Health Insurance in the Department of Health and Welfare, Iwate Prefecture Government.

Transparency declaration

The lead authors (ST and IK) affirm that the manuscript is an honest, accurate, and transparent account of the study being reported; that no important aspects of the study have been omitted; and that any discrepancies from the study as planned have been explained.

Dissemination to participants and related patient and public communities

No dissemination to participants and related patient and public communities have been organized., no dissemination to patient organizations is not applicable.

For peer review only

For peer review only - http://bmjopen.bmj.com/site/about/guidelines.xhtml

References

1. Johns Hopkins University and Medicine, USA. COVID-19 Data in Motion: Monday, March 29, 2021. 2021. https://coronavirus.jhu.edu/ （accessed 29 March 2021）.

2. Ministry of Health, Labour and Welfare, Japan. The number of positive cases for Novel Coronavirus Disease. 2021. https://www.mhlw.go.jp/content/10906000/000759133.pdf (accessed 2 June 2021).

3. Looi M-K. Covid-19: Japan declares second state of emergency as Asia struggles with virus surge. *BMJ* 2021;**372**:n141. doi: 10.1136/bmj.n141.

4. Akane O, Mitsuru O. Japan's second COVID state of emergency: Five things to know. *Nikkei Asia.* January 7 2021. https://asia.nikkei.com/Spotlight/Coronavirus/Japan-s-second-COVID-state-of-emergency-Five-things-to-know (accessed 27 March 2021).

5. Shahnazi H, Ahmadi-Livani M, Pahlavanzadeh B, *et al*. Assessing preventive health behaviors from COVID-19: a cross sectional study with health belief model in Golestan Province, Northern of Iran. *Infect Dis Poverty* 2020;**9**(1):157. doi: 10.1186/s40249-020-00776-2.

6. Chen X, Chen H. Differences in Preventive Behaviors of COVID-19 between Urban and Rural Residents: Lessons Learned from A Cross-Sectional Study in China. *Int. J. Environ. Res. Public Health* 2020;**17**(12):4437. doi: 10.3390/ijerph17124437.

7. Li S, Feng B, Liao W, *et al*. Internet Use, Risk Awareness, and Demographic Characteristics Associated With Engagement in Preventive Behaviors and Testing: Cross-Sectional Survey on COVID-19 in the United States. *J. Medical Internet Res.* 2020;**22**(6):e19782. doi: 10.2196/19782.

8. An L, Hawley S, Van Horn ML, *et al*. Development of a coronavirus social distance attitudes scale. *Patient Educ Couns.* 2020;**104**(6):1451-1459. doi: 10.1016/j.pec.2020.11.027.

For peer review only - http://bmjopen.bmj.com/site/about/guidelines.xhtml

9. Iwate Prefecture, Japan. Information on Confirmed Cases in Iwate. 2021. https://www.pref.iwate.jp/kurashikankyou/iryou/covid19/index.html (accessed 13 March 2021).

10. The microCOVID Project, San Francisco. microCOVID Project. 2020. https://www.microcovid.org (accessed 27 November 2020).

11. Lietz J, Westermann C, Nienhaus A, *et al*. The Occupational Risk of Influenza A (H1N1) Infection among Healthcare Personnel during the 2009 Pandemic: A Systematic Review and Meta-Analysis of Observational Studies. *PLoS ONE* 2016;**11**(8):e0162061. doi: 10.1371/journal.pone.0162061.

12. Marinaccio A, Boccuni F, Rondinone BM, *et al*. Occupational factors in the COVID-19 pandemic in Italy: compensation claims applications support establishing an occupational surveillance system. *Occup Environ Med* 2020;**77**(12):818-21. doi: 10.1136/oemed-2020-106844.

13. Nguyen LH, Drew DA, Graham MS, *et al*. Risk of COVID-19 among front-line health-care workers and the general community: a prospective cohort study. *Lancet Public Health* 2020;**5**(9):e475-e83. doi: 10.1016/s2468-2667(20)30164-x.

14. Mutambudzi M, Niedwiedz C, Macdonald EB, *et al*. Occupation and risk of severe COVID-19: prospective cohort study of 120 075 UK Biobank participants. *Occup Environ Med* 2020;**0**:1-8. doi: 10.1136/oemed-2020-106731.

15. Viner RM, Mytton OT, Bonell C, *et al*. Susceptibility to SARS-CoV-2 Infection Among Children and Adolescents Compared With Adults: A Systematic Review and Meta-analysis. *JAMA Pediatr* 2021;**175**(2):143-56. doi: 10.1001/jamapediatrics.2020.4573.

For peer review only - http://bmjopen.bmj.com/site/about/guidelines.xhtml

16. Yung CF, Kam K-q, Nadua KD, *et al*. Novel Coronavirus 2019 Transmission Risk in Educational Settings. *Clin. Infect. Dis.* 2020;**72**(6):1055-1058. doi: 10.1093/cid/ciaa794.

17. Junko K, Tamie S, Yasushi O. The second COVID-19 emergency status declaration for Japan: effects as of February 7, 2021. *medRxiv* 2021:2020.12.29.20248977. doi: https://doi.org/10.1101/2020.12.29.20248977

18. Centers for Disease Control and Prevention, USA. Information for Pediatric Healthcare Providers. 2020. https://www.cdc.gov/coronavirus/2019-ncov/hcp/pediatric-hcp.html (accessed 31 March 2021).

19. Vlachos J, Hertegård E, B. Svaleryd H. The effects of school closures on SARS-CoV-2 among parents and teachers. *PNAS* 2021;**118**(9):e2020834118. doi: 10.1073/pnas.2020834118.

20. Ministry of Health, Labour and Welfare, Japan. 22th meeting of the COVID-19 advisory board of Ministry of Health, Labour and Welfare. 2021. https://www.mhlw.go.jp/content/10900000/000759091.pdf (accessed 1 May 2021).

For peer review only

For peer review only - http://bmjopen.bmj.com/site/about/guidelines.xhtml

Figure Legends

Fig. 1. Flow chart of the selection of respondents.

Of 100,958 registered residents, 9,741 residents were selected for this study.

Fig. 2. Analytic models for behavioral risk trajectories.

For peer review only

For peer review only - http://bmjopen.bmj.com/site/about/guidelines.xhtml

Supplementary data

S1 Fig. Map of the study area.

Gray area represents Iwate, Japan.

S1 Text. Detailed calculation of microCOVID.

S1 Table. Weighting coefficient in items in each factor in activity and person risk.

S2 Table. Results of analysis using models for risk trajectories by multiple imputation methods (n=9,876).

S3 Table. Comparison of characteristics of the participants in the first survey and the characteristics of the whole population in the 2020 local census in Iwate.

For peer review only

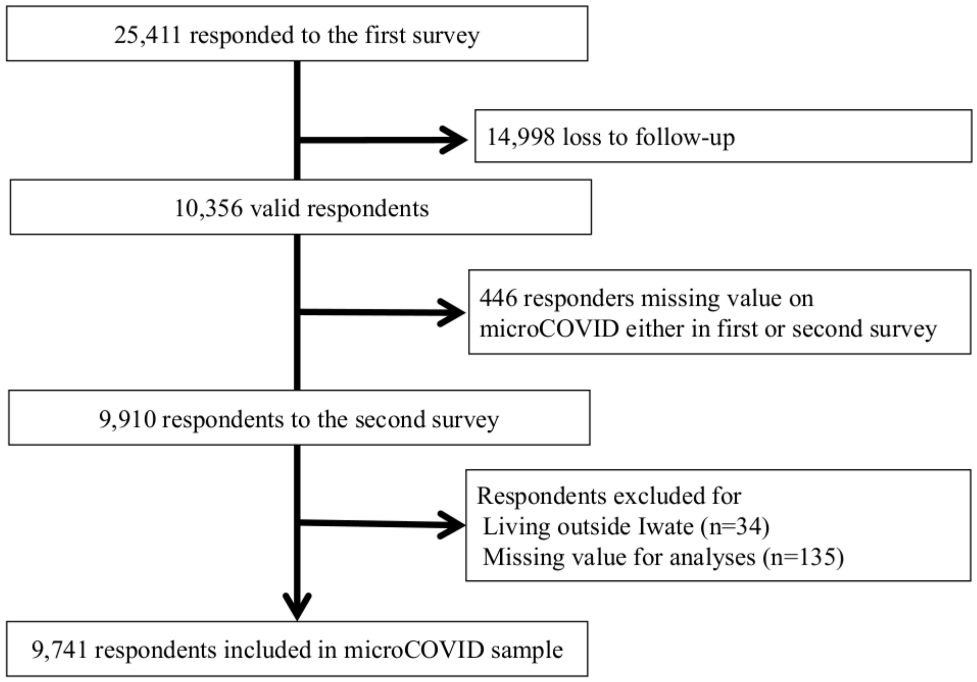

Fig. 1. Flow chart of the selection of respondents.

82x58mm (300 x 300 DPI)

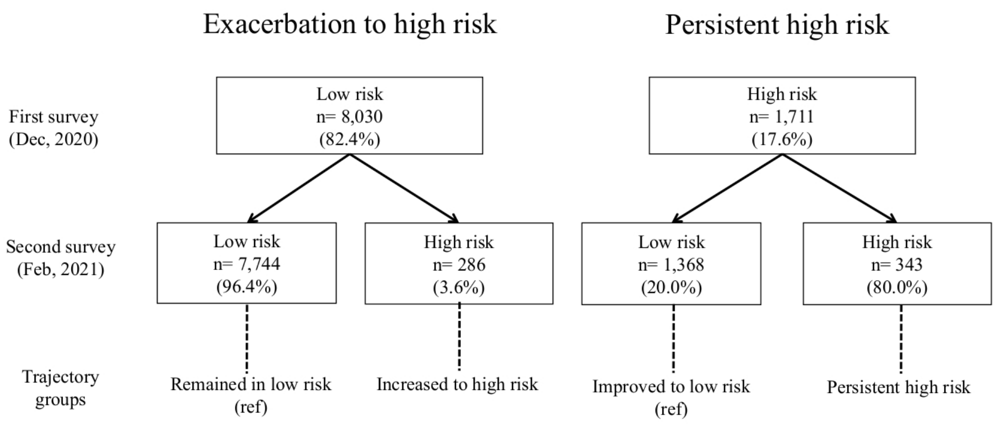

Fig. 2. Analytic models for behavioral risk trajectories.

112x50mm (300 x 300 DPI)

Supplementary data

S1 Text. Detailed calculation of microCOVID.

The microCOVID project is a calculator to measure and numerically quantify the risk of getting COVID-19 from an activity or relationship in participants' daily life based on available evidence. 1 microCOVID means a one-in-a-million chance of getting COVID. Microcovids are computed by using three major factors: activity risk, person risk, and number of people. We obtained the value by multiplying person interactions: microCOVID = Activity Risk x Number of people x Person Risk for one person.

Activity risk indicates the chance that the activity will transmit COVID from one to the other person who currently has COVID. In accordance with a previous study conducted by The Behavioral Insights Team in the UK [1], we modified some items in the questionnaire by asking respondents "did you meet anyone outside your household (or support bubble) in a risky situation in the past week?". A microCOVID value of zero was given to respondents who did not meet any person. Respondents who answered that they met someone went to the next questions asked about the details of the microCOVIDs value. Activity risk is a weighting based on coefficients how the interaction in the question is different from the reference value. Coefficients of risk are assigned to other types of interaction which differ from that reference value (if one person who has COVID, for 1 hour, indoors, unmasked, at 3 feet, there is a baseline 9% chance of transmission per hour) based on the following factors: duration of interaction, mask wearing (respondents and other persons), indoor/outdoor environment (indoor, outdoor, indoor with a HEPA filter, a train with air filtration, an airplane, a moving car with the windows rolled down, a space with one or more sides open to the outdoors), distance from each other, volume of conversation, and frequency (times a week). We calculated Activity Risk by multiplying 9%: Activity risk = coefficients x 9% (**S1 Table**).

For peer review only

With regard to the number of people, we asked respondents "how many people were there within a 5 meter radius of the scene?".

Person risk represents the chance that the other person currently has COVID based on overall prevalence in the person's area and recent behaviors of the person. Because the chance of a person having COVID depends not only on whether that person has any symptoms but also on the actions and choices of that person in the past 10 days, we used three different methods for estimating the probability of a person having COVID: a basic method, an intermediate method and an advanced method. The basic method is just an assumption that a person is "average" for their region. The probability of your friend having COVID is the probability of anyone in their geographic area having COVID. New infections in the past week were calculated as follows: new infections in the past week = reported cases x underreporting factor x delay factor. Based on that value, we estimated the person risk as follows: person risk (basic) = New infections in the past week / population in millions. The intermediate method is a quick adjustment for the risk a person being exposed at his/her workplace. The advance method involves adding up the risk of each individual activity that the person has done recently with consideration of three categories: socializing, errands, and work. In the intermediate and advanced methods, we calculated person risk by multiplying person risk (basic) by weighting coefficients in the following 12 items (**S1 Table**): "being an essential worker (working in health care, transportation or retailing)", "living alone and only going to grocery stores for shopping", "living in a closed pod of 4 people", "living in a closed pod of 10 people", "living in a closed pod of 20 people", "having gone to a bar in the past 10 days", "going to a bar frequently", "having been a patient with COVID-19", "you and other persons living with you only going to grocery stores for shopping", "other persons living with

For peer review only - http://bmjopen.bmj.com/site/about/guidelines.xhtml

48 you not being essential workers", "not have any interaction with others and other persons
49 living with you who are essential workers", and "not applicable for those items".

For peer review only

For peer review only - http://bmjopen.bmj.com/site/about/guidelines.xhtml

S1 Table. Weighting coefficient in items in each factor in activity and person risk.

Risk	Factors	Items	Coefficient
**Activity Risk**	**Respondents' mask**	No mask or inappropriate usage	1
		Polyester or polyurethane mask	0.5
		Cloth or bandanna	0.25
		Surgical mask, non-woven mask	0.25
		N95 mask	0.1
	**Other person's mask**	No mask or inappropriate usage	1
		Polyester or polyurethane mask	1
		Cloth or bandanna	1
		Surgical mask, non-woven mask	0.5
		N95 mask	0.1
	**Indoor/outdoor environment**	Indoor without ventilation	1
		Outdoor	0.05
		Indoor with a HEPA filter	0.25
		A train with air filtration	0.25
		An airplane	0.17
		A moving car with the windows rolled down	0.25
		A space with one or more sides open to the outdoors	0.25
	**Distance from each other**	< 0.3 meters apart	2

For peer review only

		About 1 meter apart	1
		2+ meters apart	0.5
		3+ meters apart	0.25
	**Volume of conversation**	Not talking	0.2
		Normal conversation	1
		Loud talking (shouting, talking over music, singing)	5
**Person Risk**	**Intermediate and Advanced Method**	Being an essential workers	2
		Living alone and only going to grocery stores for shopping	0.011
		Living in a closed pod of 4 people	0.021
		Living in a closed pod of 10 people	0.041
		Living in a closed pod of 20 people	0.075
		Having gone to a bar in the past 10 days	14.5
		Going to a bar frequently	0.9
		Having been a patient with COVID-19	1000000
		You and other persons living with you only going to grocery stores for shopping	0.014
		Other persons living with you not being essential workers	0.5
		Not have any interaction with others and other persons living with you who are essential workers	0.61
		Not applicable for those items	1

For peer review only - http://bmjopen.bmj.com/site/about/guidelines.xhtml

S2 Table. Results of analysis using models for risk trajectories by multiple imputation methods (n=9,876).

		Model for transition to high risk (n=8,113)		Model for high risk persistence (n=1,763)	
		**OR (95% CI)**	**P-value**	**OR (95% CI)**	**P-value**
**Age groups**	**Young (ref: elderly)**	1.96 (1.19 - 3.24)	0.008	1.59 (0.90 - 2.81)	0.111
	**Middle age**	1.51 (0.93 - 2.44)	0.092	1.49 (0.86 - 2.57)	0.157
**Sex**	**Women (ref: men)**	1.05 (0.80 - 1.37)	0.733	1.25 (0.91 - 1.71)	0.17
**Area**	**Inland areas (ref: coastal and mountainous areas)**	0.99 (0.75 - 1.32)	0.958	0.83 (0.62 - 1.11)	0.207
**Occupation**	**Health care workers (ref: government workers)**	2.66 (1.76 - 4.01)	<0.001	1.78 (1.17 - 2.73)	0.008
	**Service**	1.18 (0.78 - 1.78)	0.437	1.51 (0.97 - 2.35)	0.07
	**Schools**	1.17 (0.69 - 1.98)	0.569	2.62 (1.59 - 4.34)	<0.001
	**Others**	0.56 (0.35 - 0.88)	0.013	1.23 (0.72 - 2.10)	0.451
**Rate of decrease in eating out compared with that in the past year**	**Decreased by 50-70% (ref: decreased by 80% or more)**	1.22 (0.91 - 1.64)	0.183	1.03 (0.76 - 1.40)	0.83
	**Decreased by 40% or less**	1.02 (0.75 - 1.39)	0.888	1.21 (0.89 - 1.64)	0.22
**Homecoming in the New Year's holiday season**	**Yes (ref: no)**	1.04 (0.75 - 1.44)	0.809	1.17 (0.85 - 1.59)	0.334
**The first shrine visit of the year**	**Do not visit every year (ref: not visited on this year for prevention)**	1.08 (0.76 - 1.55)	0.664	1.81 (1.23 - 2.66)	0.003
	**Visited**	1.03 (0.78 - 1.35)	0.853	1.12 (0.85 - 1.49)	0.414
**Measures in the second state of emergency**	**Lower (ref: higher)**	1.59 (1.04 - 2.44)	0.032	1.28 (0.85 - 1.94)	0.244
	**Same**	0.90 (0.65 - 1.23)	0.487	0.77 (0.57 - 1.04)	0.085

Abbreviations: CI, confidence interval; OR, odds ratio

For peer review only

For peer review only - http://bmjopen.bmj.com/site/about/guidelines.xhtml

S3 Table. Comparison of characteristics of the participants in the first survey and the characteristics of the whole population in the 2020 local census in Iwate.

		2020 local census	All analytic sample in the first survey
		n (%)	n (%)
**Sex**	**Men**	585077 (48.3)	2692 (27.6)
	**Women**	627124 (51.7)	7049 (72.4)
**Age classes**	**Young**	396943 (32.7)	2765 (28.4)
	**Middle age**	315660 (26.0)	5802 (59.6)
	**Elderly**	492455 (40.6)	1174 (12.1)
	**Unknown**	7143 (0.6)	Not applicable
**Area**	**Inland areas**	928199 (76.6)	7859 (80.7)
	**Coastal and mountainous areas**	284002 (23.4)	1882 (19.3)

1. Yihan X, Mark E, Tania L, Lev T, Abigail M, Hugo H. A small number of people account for a large amount of coronavirus risk. In: team Tbi, ed. United Kingdom: The behavioural insights team, 2020.

For peer review only - http://bmjopen.bmj.com/site/about/guidelines.xhtml

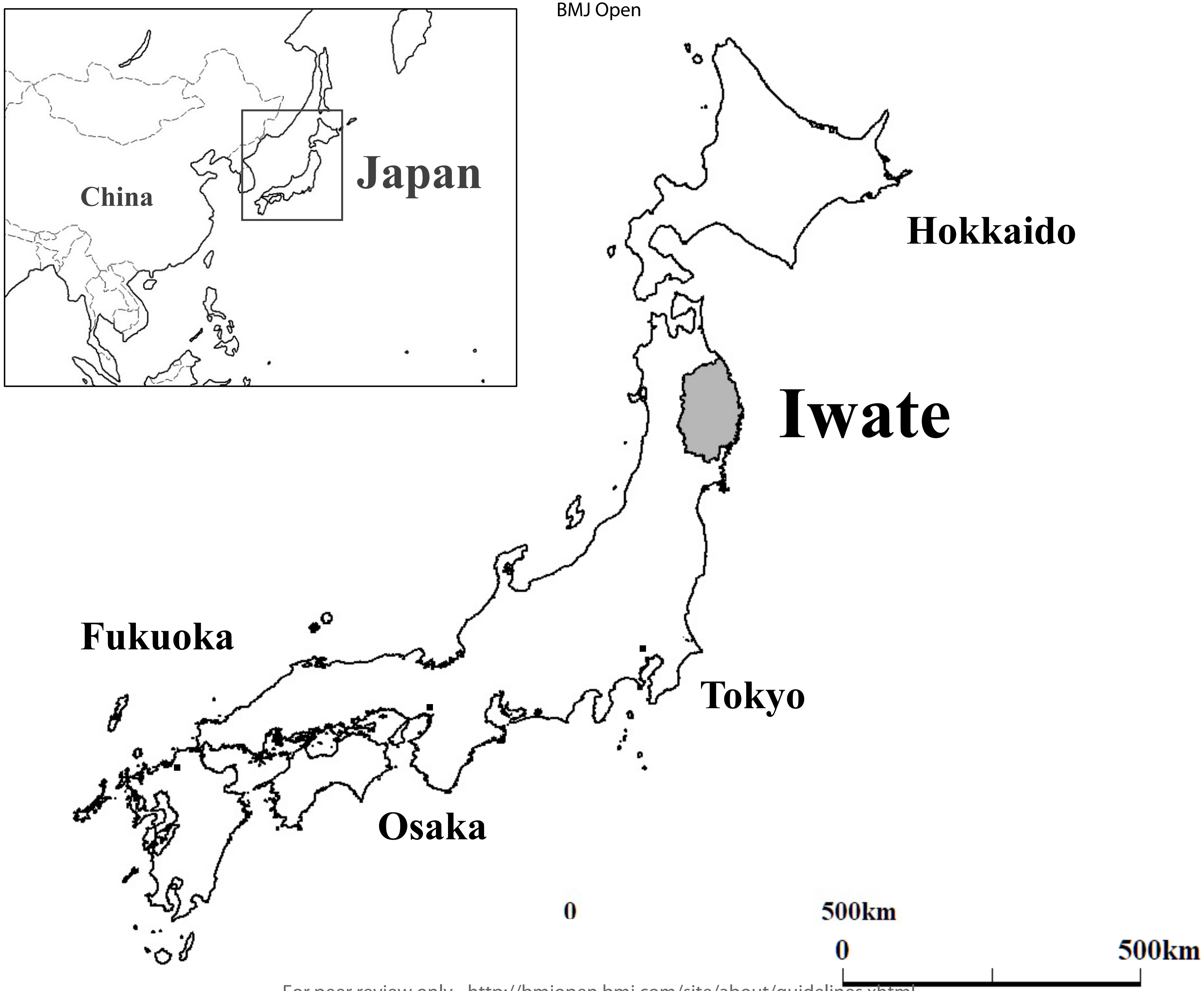
China
Japan
Hokkaido
Iwate
Fukuoka
Tokyo
Osaka
0
500km
0
500km

STROBE 2007 (v4) checklist of items to be included in reports of observational studies in epidemiology*
Checklist for cohort, case-control, and cross-sectional studies (combined)

Section/Topic	Item #	Recommendation	Reported on page #
**Title and abstract**	1	(*a*) Indicate the study's design with a commonly used term in the title or the abstract	Page 1
		(*b*) Provide in the abstract an informative and balanced summary of what was done and what was found	Page 2-3
**Introduction**			
Background/rationale	2	Explain the scientific background and rationale for the investigation being reported	Page 4-5
Objectives	3	State specific objectives, including any pre-specified hypotheses	Page 5
**Methods**			
Study design	4	Present key elements of study design early in the paper	Page 5-6
Setting	5	Describe the setting, locations, and relevant dates, including periods of recruitment, exposure, follow-up, and data collection	Page 5-6, Fig .1-2, Supplementary S1 Fig
Participants	6	(*a*) *Cohort study*—Give the eligibility criteria, and the sources and methods of selection of participants. Describe methods of follow-up<br>*Case-control study*—Give the eligibility criteria, and the sources and methods of case ascertainment and control selection. Give the rationale for the choice of cases and controls<br>*Cross-sectional study*—Give the eligibility criteria, and the sources and methods of selection of participants	Page 5-6
		(*b*) *Cohort study*—For matched studies, give matching criteria and number of exposed and unexposed<br>*Case-control study*—For matched studies, give matching criteria and the number of controls per case	Page 5-6
Variables	7	Clearly define all outcomes, exposures, predictors, potential confounders, and effect modifiers. Give diagnostic criteria, if applicable	Page 6-8
Data sources/ measurement	8*	For each variable of interest, give sources of data and details of methods of assessment (measurement). Describe comparability of assessment methods if there is more than one group	Page 6-8
Bias	9	Describe any efforts to address potential sources of bias	Page 12
Study size	10	Explain how the study size was arrived at	Page 5-6
Quantitative variables	11	Explain how quantitative variables were handled in the analyses. If applicable, describe which groupings were chosen and why	Page 6-7, Supplementary S1 Text
Statistical methods	12	(*a*) Describe all statistical methods, including those used to control for confounding	Page 8
		(*b*) Describe any methods used to examine subgroups and interactions	Page 6-8
		(*c*) Explain how missing data were addressed	Page 8

For peer review only

		(*d*) *Cohort study*—If applicable, explain how loss to follow-up was addressed<br>*Case-control study*—If applicable, explain how matching of cases and controls was addressed<br>*Cross-sectional study*—If applicable, describe analytical methods taking account of sampling strategy	Page 8
		(*e*) Describe any sensitivity analyses	Page 8
**Results**			
Participants	13*	(a) Report numbers of individuals at each stage of study—eg numbers potentially eligible, examined for eligibility, confirmed eligible, included in the study, completing follow-up, and analysed	Page 6
		(b) Give reasons for non-participation at each stage	Page 6
		(c) Consider use of a flow diagram	Page 6, Fig. 1
Descriptive data	14*	(a) Give characteristics of study participants (eg demographic, clinical, social) and information on exposures and potential confounders	Page 9, Table. 1
		(b) Indicate number of participants with missing data for each variable of interest	Fig. 1
		(c) *Cohort study*—Summarise follow-up time (eg, average and total amount)	Page 5-6, Fig. 2
Outcome data	15*	*Cohort study*—Report numbers of outcome events or summary measures over time	Page 6-7, Fig. 2
		Case-control study—Report numbers in each exposure category, or summary measures of exposure	
		Cross-sectional study—Report numbers of outcome events or summary measures	
Main results	16	(*a*) Give unadjusted estimates and, if applicable, confounder-adjusted estimates and their precision (eg, 95% confidence interval). Make clear which confounders were adjusted for and why they were included	Page 12-13, Table 2
		(*b*) Report category boundaries when continuous variables were categorized	Page 6-7, Supplementary S1 Text
		(*c*) If relevant, consider translating estimates of relative risk into absolute risk for a meaningful time period	
Other analyses	17	Report other analyses done—eg analyses of subgroups and interactions, and sensitivity analyses	Page 14, S2 Table
**Discussion**			
Key results	18	Summarise key results with reference to study objectives	Page 14
Limitations	19	Discuss limitations of the study, taking into account sources of potential bias or imprecision. Discuss both direction and magnitude of any potential bias	Page 17
Interpretation	20	Give a cautious overall interpretation of results considering objectives, limitations, multiplicity of analyses, results from similar studies, and other relevant evidence	Page 14-16
Generalisability	21	Discuss the generalisability (external validity) of the study results	Page 17
**Other information**			
Funding	22	Give the source of funding and the role of the funders for the present study and, if applicable, for the original study on which the present article is based	Page 18

For peer review only

*Give information separately for cases and controls in case-control studies and, if applicable, for exposed and unexposed groups in cohort and cross-sectional studies.
Note: An Explanation and Elaboration article discusses each checklist item and gives methodological background and published examples of transparent reporting. The STROBE checklist is best used in conjunction with this article (freely available on the Web sites of PLoS Medicine at http://www.plosmedicine.org/, Annals of Internal Medicine at http://www.annals.org/, and Epidemiology at http://www.epidem.com/). Information on the STROBE Initiative is available at www.strobe-statement.org.

For peer review only

BMJ Open

Increase in people's behavioral risks for contracting COVID-19 during the 2021 New Year holiday season: Longitudinal survey of the general population in Japan

Journal:	*BMJ Open*
Manuscript ID	bmjopen-2021-054770.R1
Article Type:	Original research
Date Submitted by the Author:	30-Nov-2021
Complete List of Authors:	Takahashi, Shuko; Iwate Prefecture Government, Department of Health and Welfare; Iwate Medical University<br>Yamada, Shohei; Iwate Prefecture Government, Department of Health and Welfare<br>Sasaki, Satoshi; Iwate Prefecture Government, Department of Health and Welfare<br>Minato, Yoichi; Iwate Prefecture Government, Department of Health and Welfare<br>Takahashi, Naomi; Iwate Medical University Faculty of Medicine Graduate School of Medicine, Department of Hygiene and Preventive Medicine<br>Kudo, Keiichiro; Iwate Prefecture Government, Department of Health and Welfare<br>Masaru, Nohara; Iwate Prefecture, Department of Health and Welfare<br>Kawachi, Ichiro; Harvard University T H Chan School of Public Health, Department of Society Human Development and
**Primary Subject Heading**:	Epidemiology
Secondary Subject Heading:	Infectious diseases, Public health, Global health, Health policy, Sociology
Keywords:	COVID-19, EPIDEMIOLOGY, Health policy < HEALTH SERVICES ADMINISTRATION & MANAGEMENT, Risk management < HEALTH SERVICES ADMINISTRATION & MANAGEMENT, Public health < INFECTIOUS DISEASES

SCHOLARONE™
Manuscripts

I, the Submitting Author has the right to grant and does grant on behalf of all authors of the Work (as defined in the below author licence), an exclusive licence and/or a non-exclusive licence for contributions from authors who are: i) UK Crown employees; ii) where BMJ has agreed a CC-BY licence shall apply, and/or iii) in accordance with the terms applicable for US Federal Government officers or employees acting as part of their official duties; on a worldwide, perpetual, irrevocable, royalty-free basis to BMJ Publishing Group Ltd ("BMJ") its licensees and where the relevant Journal is co-owned by BMJ to the co-owners of the Journal, to publish the Work in this journal and any other BMJ products and to exploit all rights, as set out in our licence.

The Submitting Author accepts and understands that any supply made under these terms is made by BMJ to the Submitting Author unless you are acting as an employee on behalf of your employer or a postgraduate student of an affiliated institution which is paying any applicable article publishing charge ("APC") for Open Access articles. Where the Submitting Author wishes to make the Work available on an Open Access basis (and intends to pay the relevant APC), the terms of reuse of such Open Access shall be governed by a Creative Commons licence – details of these licences and which Creative Commons licence will apply to this Work are set out in our licence referred to above.

Other than as permitted in any relevant BMJ Author's Self Archiving Policies, I confirm this Work has not been accepted for publication elsewhere, is not being considered for publication elsewhere and does not duplicate material already published. I confirm all authors consent to publication of this Work and authorise the granting of this licence.

For peer review only

Increase in people's behavioral risks for contracting COVID-19 during the 2021 New Year holiday season: Longitudinal survey of the general population **in Japan**

Shuko Takahashi [a, b] *, Shohei Yamada [a], Satoshi Sasaki [a], Yoichi Minato [a], Naomi Takahashi [c], Keiichiro Kudo [a], Masaru Nohara [a], and Ichiro Kawachi [d]

[a] Office of Medical Policy, Department of Health and Welfare, Iwate Prefecture Government, 10-1 Uchimaru, Morioka, Iwate 020-8570, Japan

[b] Division of Medical Education, Iwate Medical University, 1-1-1, Idaidori, Yahaba-cho, Shiwa-gun, Iwate 028-3694, Japan

[c] Graduate School of Medicine, Faculty of Medicine, Iwate Medical University, School of Medicine, 1-1-1, Idaidori, Yahaba-cho, Shiwa-gun, Iwate 028-3694, Japan

[d] Harvard. T.H. Chan School of Public Health, 401 Park Drive, Boston MA 02215, USA

* Corresponding author. Shuko Takahashi, MD, PhD. Office of Medical Policy, Department of Health and Welfare, Iwate Prefecture Government, 10-1 Uchimaru, Morioka, Iwate 020-8570, Japan.

Tel: +81-19-651-3111, Fax: +81-19-651-3111, E-mail: shutakahashi-iwt@umin.ac.jp

Keywords; COVID-19, Epidemiology, Health policy, Risk management, Public health

For peer review only

For peer review only - http://bmjopen.bmj.com/site/about/guidelines.xhtml

Abstract

Objectives: There has been no study in Japan on the predictors of risk for acquiring SARS-CoV-2 infection based on people's behavior during the COVID-19 pandemic. The aim of this study was to document changes in risk behavior during the New Year's holiday season in 2021 and to identify factors associated with high-risk behavior for infection using a quantitative assessment tool.

Design: A longitudinal survey.

Setting: Multiphasic health check-ups for the general population in Iwate Prefecture.

Participants: Serial cross-sectional data were obtained using rapid online surveys of residents in Iwate Prefecture from December 4 to 7 in 2020 (baseline survey) and from February 5 to 7 in 2021 (followup survey). The data in those two surveys was available for a total of 9,741 participants.

Main outcome measures: We estimated each individual's risk of acquiring SARS-CoV-2 infection based on the microCOVID calculator. We defined four trajectories of individual risk behaviors based on the probabilities of remaining at low risk, increasing to high risk, improving to low risk, and persistence of high risk.

Results: Among people in the low risk group in the first survey, 3.6% increased to high risk, while high risk persisted in 80.0% of people who were in the high risk group at baseline. While health care workers were significantly more likely to be represented in both the increasing risk and persistently high risk group, workers in the education setting were also associated with persistence of high risk (OR [95% confidence interval], 2.58 [1.52–4.39]; P <0.001).

For peer review only - http://bmjopen.bmj.com/site/about/guidelines.xhtml

CONCLUSIONS: In determining counter-measures against COVID-19 (as well as future outbreaks), health officials should take into account population changes in behavior during large-scale public events.

Strengths and limitations of this study

1. Risk behaviors associated with contracting COVID-19 were assessed in a large sample of the general population living in one Japanese prefecture.
2. Longitudinal assessments were conducted at two time points, straddling the 2021 holiday season.
3. The brief, online survey approach allowed for rapid, real time tracking of population changes in behavior.
4. A limitation of the study was the low response rate to the baseline survey (25%) as well as the follow-up survey (40%), although the direction of the bias is likely to be conservative.

For peer review only

For peer review only - http://bmjopen.bmj.com/site/about/guidelines.xhtml

Introduction

Cases of COVID-19 spread worldwide soon after the first case was identified in China in 2019. The worldwide death toll from COVID-19 as of November 19, 2021 is 5,139,910 [1]. The first case of COVID-19 in Japan was confirmed in Kanagawa on January 16, 2020, and cases then spread from urban areas to rural areas in Japan.

In December 2020, Japan experienced a third wave which was much larger than the second wave, peaking at 8,045 new cases on January 8, 2021 (compared to 1,575 cases at the peak of the second wave on July 31, 2020) [2]. The Japanese government declared a second state of emergency for Tokyo and its surrounding prefectures on January 7, 2021, and several measures were implemented including shortening the opening hours for bars and restaurants and encouraging tele-work to decrease the number of commuters by 70% [3].

One of the reasons for the increase in the number of COVID-19 cases in Japan was the movement of people during the traditional New Year holiday. New Year's Day is the most important holiday in Japan, when many people return to their hometowns to spend time with their family, relatives or local friends. The typical number of days that people take off during the New Year's holiday is about one week (seven days) from December 28 to January 3. Although there is a decrease in the movement of people because schools, businesses and workplaces are closed for several days, large crowds of people gather to pray at local shrines or temples on New Year's Day (called "Hatsumode" in Japanese) [4].

The Japanese government opted to not restrict the movement of people during the period before and after New Year's Day. Some people also ignored the government's request to refrain from unnecessary outings, while other people continued to take preventive measures against infection. However, the number of people who ignored government directives is not known. Studies have shown that there are variations in the tendency to adopt protective

For peer review only

For peer review only - http://bmjopen.bmj.com/site/about/guidelines.xhtml

measures such as social distancing based upon on people's age, gender, socioeconomic characteristics (educational attainment, occupation), and political ideology [5-8]. However, there were a few tools to examine people's daily behavior to quantify the risk of being infected based on people's daily activities. In addition, the extent of contagion differs with regional conditions, as well as population mobility during special events such as national holidays. In order to establish effective interventions during large-scale public events, there is a need to identify high-risk groups using an objective tool.

The aim of our study was to determine different groups of individuals based on their trajectories of risk behavior during the New Year holiday season and to determine factors associated with behavior with a high risk for COVID-19 using a quantitative risk assessment tool, "microCOVID".

Materials and methods

Study area

Iwate Prefecture is located in the northeastern part of Japan (about 500 km from Tokyo) with a population of about 1.2 million (**S1 Fig**). The total number of COVID-19 cases as of November 19, 2021 was 3487 with 53 cases of COVID-19-related deaths reported[9]

Data

Rapid online surveys of residents in Iwate Prefecture were conducted by the Office of Medical Policy in the Department of Health and Welfare in Iwate Prefecture using a popular social network platform called LINE (LINE Corporation, Tokyo, Japan). By using the LINE app, Iwate Prefecture has been providing information about COVID-19 every day since the

For peer review only - http://bmjopen.bmj.com/site/about/guidelines.xhtml

beginning of the pandemic, including information on the number of new cases and characteristics of patients. A series of cross-sectional surveys were started in December 2020 to investigate people's behavior in order to prevent infection and promote health consciousness. The surveys have been conducted every two month. An online questionnaire was administered to a total of 100,958 people who had registered by the time of the baseline survey. We sent out notification about the survey via the LINE platform on December 4, 2020, and 25,411 individuals answered from December 4 to 7 (participants rate, 25.2%). We conducted a second survey of registered people from February 5 to 7, 2021. We analyzed the results of these two surveys. After excluding individuals who were lost to follow-up in the second survey (14,998), those who were missing data needed for the calculation of microCOVID scores either in first or second survey (n=436), those who live outside Iwate (n=34) and those who had other missing values for analyses (n=135), data were available for a total of 9,741 participants (**Fig. 1**).

Ethics approval statement

The surveys were conducted in accordance with applicable Japanese law and Iwate Prefectural Government policy. Since this study was a secondary analysis using anonymized data from Iwate Prefecture, the study was exempt from IRB review.

Outcome

We applied a weighting system to calculate the level of behavioral risk for acquiring COVID-19 infection, called microCOVID [10]. MicroCOVID is a calculator to numerically quantify the risk of getting COVID-19 from daily activities. 1.0 microCOVID is equivalent to a one-in-a-million chance of getting COVID. Microcovids are computed by using three major

For peer review only - http://bmjopen.bmj.com/site/about/guidelines.xhtml

factors: activity risk, person risk, and number of people with whom an individual interacts with (**S1 Text**). We obtained the microCOVID value for each person whereby the score = activity risk x number of people x person risk.

Activity risk indicates the chance that an activity will transmit COVID from a person who currently is infected with COVID. According to microCOVID, the transmission risk is estimated to be "about 9% per hour" from spending more than 10 minutes indoors or in close proximity with an unmasked person who is COVID-positive. Coefficients of risk are assigned to other types of interaction which differ from that reference value. The coefficients were calculated based on the following factors: duration of interaction, mask wearing (respondents and other persons), indoor/outdoor environment, distance from each other, volume of conversation, and frequency (times a week) (**S1 Table**). With regard to the number of people, we asked respondents "how many people were there within a 5 meter radius of the scene?". Person risk represents the probability that a random person is currently infected with COVID based on the overall prevalence in the person's area as well as the recent behaviors of the person (**S1 Table**). We classified each individual's risk level into low-risk versus high-risk for acquiring infection (low-risk group, ≤20 microCOVIDs; high-risk group, >20 microCOVIDs).

Covariates

Age, sex, municipality, occupation, frequency of eating out compared with the past year, returning home during the New Year holiday, visits to the local shrine/temple were included as independent variables in the analysis. Participants were divided into three age categories: young (people less than 39 years of age), middle age (people of 40 to 59 years of age) and elderly (people aged 60 years or older). Residential areas were classified into inland versus

For peer review only - http://bmjopen.bmj.com/site/about/guidelines.xhtml

coastal/mountainous based on the geography of Iwate prefecture. Occupations were assessed by asking “what is your current job?”. Participants were divided into five groups: health care workers, workers in service industries (e.g., transportation, customer-facing occupations in the retail/hospitality sector, office workers), education sector (teachers or students), government workers, and all others (workers in manufacturing, farmers/agricultural workers, workers in other jobs, or unemployed). The frequency of eating out compared with the past year was classified into three groups: "decreased by 80% or more", "decreased by 50-70%" and “decreased by 40% or less”. With regard to the first shrine visit of the year, participants were divided into three categories: “never observe the ceremony”, “visited” and “did not visit this year as a preventive measure”. We asked respondents about the extent of preventive measures adopted during the second state of emergency compared with that in the first one. The participants were divided into three groups: lower (lower degree or no preventive measure), same (same as the first one) and higher (higher degree).

Statistical analyses

For analysis of the trajectories of behavior risks, we calculated the risk scores for each individual in the first survey and that in the second survey. Baseline characteristics were compared for the low-risk and high-risk microCOVID groups in the second survey using the chi-squared test. Logistic regression analyses was conducted to identify the characteristics related to high-risk behavior. We conducted analyses separated by low risk and high risk in the first survey to determine the predictors of each type of trajectory. We imputed missing covariate data by multiple imputation using Markov Chain Monte Carlo method, creating five imputed datasets. We used the Statistical Package for Social Sciences (SPSS) software program

For peer review only

version 25.0 (IBM, Chicago, IL, USA) for all analyses. All statistical tests described were two-sided, and analysis items with P-values <0.05 were considered statistically significant.

Patient and public involvement

No funding was available for patient or public involvement in this project. No patients were involved in setting the research question or the outcome measures. Patients were not invited in the design, or conduct, or reporting, or dissemination plans of our research.

Results

Fig. 2 shows trajectories of behavioral risks from the first survey to the second survey. In December 2020, 17.6% of the respondents were classified into the high-risk behavior category. Two months after the first survey (straddling the New Year holidays), 3.6% of the low-risk individuals in the initial survey had transitioned to high risk, while 80.0% of the individuals in the high-risk behavior category in the baseline survey remained in the high-risk group. The percentage of participants in the low-risk group was higher in the second survey than in the first survey, reflecting the decline in overall prevalence in Iwate Prefecture by the time of the followup survey.

Table 1 shows the baseline characteristics of participants in the low-risk group and high-risk group at the baseline survey. Individuals in the low-risk group at the baseline survey who later transitioned to high risk included a higher proportion of young people compared to individuals who remained in the low-risk group. Health care workers were also over-represented in the group who transitioned to high risk (35.0%) compared to those who remained at low risk (16.5%, P <0.001). The high risk group at baseline also included people in education (teachers and students), as well as individuals who reported never observing traditional visits to shrines/temples during New Year.

For peer review only

For peer review only - http://bmjopen.bmj.com/site/about/guidelines.xhtml

Table 1. Baseline characteristics of participants in the first survey (n=9,741).

		Low risk in the first survey (n=8030)				High risk in the first survey (n=1711)			
		Remained in low risk (n=7744)	Increased to high risk (n=286)			Improved to low risk (n=1368)	Persistent high risk (n=343)		
		n (%)	n (%)	*P* value		n (%)	n (%)	*P* value	
Age groups	Young	2127 (27.5)	108 (37.8)	<0.001	*	414 (30.3)	116 (33.8)	0.085	
	Middle age	4577 (59.1)	161 (56.3)			852 (62.3)	212 (61.8)		
	Elderly	1040 (13.4)	17 (5.9)			102 (7.5)	15 (4.4)		
Sex	Women	5476 (70.7)	211 (73.8)	0.263		1078 (78.8)	284 (82.8)	0.100	
Area	Inland areas	6250 (80.7)	232 (81.1)	0.863		1107 (80.9)	270 (78.7)	0.357	
Occupation	Health care workers	1274 (16.5)	100 (35.0)	<0.001	*	519 (37.9)	146 (42.6)	0.001	*
	Service	2295 (29.6)	87 (30.4)			385 (28.1)	91 (26.5)		
	Schools	600 (7.7)	24 (8.4)			116 (8.5)	48 (14.0)		
	Others	2671 (34.5)	45 (15.7)			167 (12.2)	30 (8.7)		
	Government workers	904 (11.7)	30 (10.5)			181 (13.2)	28 (8.2)		
Rate of decrease in eating out compared with that in the past year	Decreased by 80% or more	4517 (58.3)	159 (55.6)	0.378		781 (57.1)	183 (53.4)	0.343	
	Decreased by 50-70%	1673 (21.6)	60 (21.0)			296 (21.6)	86 (25.1)		
	Decreased by 40% or less	1554 (20.1)	67 (23.4)			291 (21.3)	74 (21.6)		
Homecoming in the New Year's holiday season	Yes	1189 (15.4)	50 (17.5)	0.328		231 (16.9)	70 (20.4)	0.126	
The first shrine visit of the year	Do not visit every year	1135 (14.7)	43 (15.0)	0.954		172 (12.6)	70 (20.4)	0.001	*
	Visited	3809 (49.2)	142 (49.7)			734 (53.7)	174 (50.7)		
	Did not visit this year for prevention	2800 (36.2)	101 (35.3)			462 (33.8)	99 (28.9)		
Measures in the second state of emergency	Lower	741 (9.6)	43 (15.0)	0.007	*	155 (11.3)	59 (17.2)	0.003	*
	Same	5345 (69.0)	180 (62.9)			933 (68.2)	204 (59.5)		

For peer review only

For peer review only - http://bmjopen.bmj.com/site/about/guidelines.xhtml

**Higher**	1658 (21.4)	63 (22.0)		280 (20.5)	80 (23.3)	

Categorical variables are presented as number of cases (%).

P values were calculated using the chi-squared test for categorical variables.

[*] Statistically significant differences between two groups.

For peer review only

209 **Table 2** summarizes the predictors of the trajectories of behavioral risks in the two models. With regard to transitioning to high risk, younger individuals as well as individuals with practicing a lower degree of preventive measures during the second state of emergency had significantly higher odds ratios (ORs). With regard to occupation, both health care workers and people in educational settings were more likely to be in the group with persistent high risk (OR [95% confidence interval (CI)], 2.58 [1.52–4.39]; P <0.001). Results were similar in the analyses with multiple imputation for missing data (n= 9,876) (**S2 Table**).

For peer review only

Table 2. Results of analysis using models for risk trajectories (n=9,741)

		Model for transition to high risk (n=8030)		Model for high risk persistence (n=1711)	
		OR (95% CI)	P-value	OR (95% CI)	P-value
Age groups	Young (ref: elderly)	2.22 (1.29 - 3.79)	0.004	1.54 (0.84 - 2.80)	0.16
	Middle age	1.60 (0.96 - 2.69)	0.073	1.50 (0.84 - 2.68)	0.166
Sex	Women (ref: men)	1.01 (0.76 - 1.33)	0.952	1.26 (0.91 - 1.74)	0.169
Area	Inland areas (ref: coastal and mountainous areas)	1.07 (0.79 - 1.46)	0.662	0.86 (0.63 - 1.16)	0.309
Occupation	Health care workers (ref: government workers)	2.49 (1.62 - 3.82)	<0.001	1.85 (1.18 - 2.90)	0.007
	Service	1.16 (0.76 - 1.78)	0.502	1.55 (0.97 - 2.47)	0.067
	Schools	1.11 (0.64 - 1.93)	0.709	2.58 (1.52 - 4.39)	<0.001
	Others	0.55 (0.34 - 0.89)	0.016	1.20 (0.68 - 2.12)	0.525
Rate of decrease in eating out compared with that in the past year	Decreased by 50-70% (ref: decreased by 80% or more)	0.98 (0.72 - 1.34)	0.918	1.24 (0.92 - 1.68)	0.158
	Decreased by 40% or less	1.25 (0.92 - 1.68)	0.151	1.04 (0.76 - 1.43)	0.8
Homecoming in the New Year's holiday season	Yes (ref: no)	1.06 (0.77 - 1.46)	0.736	1.18 (0.86 - 1.61)	0.299
The first shrine visit of the year	Do not visit every year (ref: not visited on this year for prevention)	1.05 (0.73 - 1.51)	0.805	1.83 (1.28 - 2.61)	0.001
	Visited	1.02 (0.79 - 1.33)	0.871	1.09 (0.83 - 1.44)	0.536
Measures in the second state of emergency	Lower (ref: higher)	1.62 (1.08 - 2.43)	0.021	1.28 (0.85 - 1.91)	0.233
	Same	0.92 (0.69 - 1.24)	0.592	0.75 (0.56 - 1.01)	0.056

Abbreviations: CI, confidence interval; OR, odds ratio

For peer review only

Discussion

We evaluated the predictors of high-risk behavior for SARS-CoV-2 infection during the Japanese New Year's holiday season in one prefecture. Overall, only a small percentage (<4%) of individuals transitioned from low risk (before the holidays) to high risk. However, the majority of individuals (80%) who were in the high risk behavior group at baseline remained high risk at follow-up.

The characteristics of individuals transitioning to high-risk behavior included younger age, people working in the health care sector, as well as individuals who admitted to practicing a lower degree of preventive measures during the state of emergency. Risk factors for persistently high risk were mainly based on occupation, viz. working in the health care sector and education sector.

Previous studies have examined factors associated with risk behaviors during COVID-19 [11-13]. Factors related to preventive knowledge and behavior for COVID-19 have been examined in several studies [5-8]. Shahnazi et al. examined the associations between preventive behaviors for COVID-19 and health belief factors, fatalistic beliefs and demographic factors among 750 Iranian adults during the period from March 11 to 16 in 2020. They showed that the adoption of preventive behavior was higher in women than men and higher in urban dwellers than rural dwellers [5]. Chen et al examined the differences between preventive behaviors for COVID-19 of urban and rural residents in China (n=1591) from January 31 to February 4 in 2020 [6]. Their results showed that rural residents were less likely to practice preventive behaviors. Li et al. examined the associations of Internet use, risk awareness, and demographic characteristics with engagement in preventative behaviors in the U.S. (n=979) from April 10 to April 14 in 2020 [7]. They showed that woman and older participants were more likely to adopt preventive behavior, and conversely more educated participants were

For peer review only

less likely to adopt preventive behavior. An et al. investigated characteristics related to positive and negative attitudes towards social distancing as a preventive measure in the U.S. (n=1074) in May 2020 [8]. They showed that female sex, older ages (65 years or older) and Democratic political party support were associated with a more positive attitude toward social distancing. Our study adds to the literature by tracking changes in population behavior during a national public holiday.

Identifying high risk occupational settings for acquiring COVID-19 infection remains an important goal for policy, e.g., prioritizing vaccinations. Health care work was recognized as a high-risk occupation during the 2009 influenza A (H1N1) pandemic [14]. Occupational risk in the current COVID-19 pandemic have been examined in several studies [15-17]. Nguyen et al. showed that the hazard ratio of the risk for COVID-19 was 3.40 [3.37-3.43] among front-line health-care workers [16]. Mutambudzi et al. reported that the risk ratio for testing positive for COVID-19 or death caused by COVID-19 was 7.43 [5.52-10.0] among healthcare workers [17]. These results are consistent with our results showing that health care workers were over-represented in both the persistently high risk group as well as the group transitioning from low to high risk. Although we could not conduct a study to validate the assessment tool due to an insufficient number of COVID-19 patients (n=15) among the respondents, an ecological survey by occupation that was conducted after the baseline survey showed that the proportion of COVID-19 infections among health care workers was high in Iwate prefecture.

Interestingly, people in the education sector had substantially higher ORs for high risk persistence even though they did not gather in schools for three weeks during the winter vacation. After our investigation period, which included 2 weeks after the second survey during the period from December 8, 2020 to February 21, 2021, about 14% of the total

For peer review only

For peer review only - http://bmjopen.bmj.com/site/about/guidelines.xhtml

number of COVID19 patients was reported to be among teachers and school-age children in Iwate. A meta-analysis showed that children and adolescents have lower susceptibility to SARS-CoV-2; the pooled odds ratio of being an infected contact in children compared with that in adults was 0.56 [0.37-0.85] [18]. A study on contact-tracing showed a lower level of transmission from index cases of children or teachers [19]. In those studies, the susceptibility to and transmission of SARS-CoV-2 in children or adolescents were compared to those in adults, and there was no comparison of the risks for teachers and students with the risks for other workers. Nguyen et al. also reported that social and education workers had a significantly higher risk for COVID-19 than did non-essential workers in the U.K and the U.S. [16]. The government in Japan closed schools at the start of the first wave of COVID-19 but did not request schools to close in the second and third waves, possibly based on the prediction of a low rate of infections in schools and less severe cases of COVID-19 in children [20]. Although fewer cases of infection and death have been reported in children and adolescents [21], a study conducted in Sweden showed that parents, teachers and partners exposed to open schools had significantly higher ORs than those in closed schools [22].

Initially, we hypothesized that people who made visits to shrines during the holidays (i.e., mingling with crowds) would have high risk for SARS-CoV-2 infection. In fact, our study did not suggest increased risk, possibly because of near universal mask-wearing, and the fact that praying at shrines & temples occurs outdoors. On the other hand, although there are some other risky behaviors that typically occur during the New Year holiday season in Japan, we could not collect detailed information on these events, e.g., “Bonen-kai” (end of year parties in workplaces) [23], and large indoor gatherings of extended families during the New Year.

For peer review only - http://bmjopen.bmj.com/site/about/guidelines.xhtml

Limitations

The present study had several limitations. First, we did not ask questions correlated with healthy behaviors such as educational attainment or household income. We also did not ask questions about job titles, which might influence the risk of exposure to potentially high-risk environments. Second, there may have been selection bias since the respondents were limited to people registered in a health information program about COVID-19 maintained by the Iwate Prefectural government. We compared characteristics of the participants at baseline (December 2020) with characteristics of the whole population in Iwate in 2020 (**S3 Table**). While the percentages of young adults and elderly were smaller in our study than in the census data, the percentages of middle-aged people and women were higher in our study than in the census data (**S3 Table**). There was a large percentage of older women in the participants in our study. We re-ran the analyses using survey weights based on demographic characteristics obtained from the Census, including sex, age group, and residential area (**S4 Table)**. In weighted analyses, younger individuals, health care workers, and people living in inland areas continued to show significantly higher ORs for transitioning to high risk. On the other hand the ORs for persistently high risk became statistically non-significant for health care workers and people in educational settings. Third, only 40% of the participants in the initial survey participated in the second survey. We compared baseline characteristics of the non-participants (n= 15,055) and participants (n=10,356) at the second survey (**S5 Table**). The percentage of younger men, people living in the inland area as well as health care workers was higher in non-participants compared to participants. Based on previous studies, people who respond to surveys about COVID-19 tend to have a higher level of consciousness about avoiding exposure/infection compared to non-respondents. Thus, we believe the direction of our attrition bias is conservative, i.e., we may have under-estimated the increase

For peer review only - http://bmjopen.bmj.com/site/about/guidelines.xhtml

in high risk behavior. Despite these limitations, the important strengths of our study were the large sample of participants who were followed at two different time points and the determination of predictors of risky behavior for COVID-19 during the New Year holiday season. This enabled us to determine trajectories of high-risk behaviors straddling a large-scale public event.

Conclusion

Health care workers and people in the education sector (teachers and pupils) were found to have the highest behavioral risk during the New Year holiday season in Japan. Our results offer some clues for who should be prioritized for COVID19 vaccination as the program is rolled out in Japan.

Acknowledgments

The authors would like to thank the members in the Office of Medical Policy in the Department of Health and Welfare as well as the health care workers who contributed to active epidemiological investigation. The work was also supported by JSPS KAKENHI Grant Numbers JP20K18858. The funders had no role in the study design, data collection and analysis, decision to publish, or preparation of the manuscript.

Contributors

All authors have participated in the concept and design, interpretation of data, critical revision and drafting or revising of the manuscript. ST: funding acquisition,

For peer review only

conceptualization, formal analysis and writing – original draft. SY: verified the underlying data, data curation, resources and writing – review & editing. SS and YM: investigation, project administration and writing ‐ review & editing. NT: investigation and writing – review & editing. KK and MN: project administration, resources, supervision and writing – review & editing. IK: methodology, supervision, validation and writing – review & editing.

Funding

The work was also supported by JSPS KAKENHI Grant Numbers JP20K18858. The funders had no role in the study design, data collection and analysis, decision to publish, or preparation of the manuscript.

Competing Interests

The authors indicated no potential conflicts of interest.

Ethical approval

The surveys were conducted in accordance with applicable Japanese law and Iwate Prefectural Government policy. Since this study was a secondary analysis using anonymized data from Iwate Prefecture, the study was exempt from IRB review.

Data sharing

The data that support the findings of this study are available from corresponding author (Dr. Shuko Takahashi) and Division of the Health and National Health Insurance in the Department of Health and Welfare, Iwate Prefecture Government but restrictions apply to the

For peer review only

For peer review only - http://bmjopen.bmj.com/site/about/guidelines.xhtml

availability of these data, which were used under license for the current study, and so are not publicly available. Data are however available from the authors upon reasonable request and with permission of Division of the National Health Insurance in the Department of Health and Welfare, Iwate Prefecture Government.

Transparency declaration

The lead authors (ST and IK) affirm that the manuscript is an honest, accurate, and transparent account of the study being reported; that no important aspects of the study have been omitted; and that any discrepancies from the study as planned have been explained.

Dissemination to participants and related patient and public communities

No dissemination to participants and related patient and public communities have been organized, no dissemination to patient organizations is not applicable.

References

1. Johns Hopkins University and Medicine, USA. COVID-19 Data in Motion: Monday, March 29, 2021. 2021. https://coronavirus.jhu.edu/ (accessed 19 November 2021).

2. Ministry of Health, Labour and Welfare, Japan. The number of positive cases for Novel Coronavirus Disease. 2021. https://www.mhlw.go.jp/content/10906000/000759133.pdf (accessed 2 June 2021).

For peer review only - http://bmjopen.bmj.com/site/about/guidelines.xhtml

3. Looi M-K. Covid-19: Japan declares second state of emergency as Asia struggles with virus surge. BMJ 2021;372:n141. doi: 10.1136/bmj.n141.

4. Akane O, Mitsuru O. Japan's second COVID state of emergency: Five things to know. Nikkei Asia. January 7 2021. https://asia.nikkei.com/Spotlight/Coronavirus/Japan-s-second-COVID-state-of-emergency-Five-things-to-know (accessed 27 March 2021).

5. Shahnazi H, Ahmadi-Livani M, Pahlavanzadeh B, et al. Assessing preventive health behaviors from COVID-19: a cross sectional study with health belief model in Golestan Province, Northern of Iran. Infect Dis Poverty 2020;9(1):157. doi: 10.1186/s40249-020-00776-2.

6. Chen X, Chen H. Differences in Preventive Behaviors of COVID-19 between Urban and Rural Residents: Lessons Learned from A Cross-Sectional Study in China. Int. J. Environ. Res. Public Health 2020;17(12):4437. doi: 10.3390/ijerph17124437.

7. Li S, Feng B, Liao W, et al. Internet Use, Risk Awareness, and Demographic Characteristics Associated With Engagement in Preventive Behaviors and Testing: Cross-Sectional Survey on COVID-19 in the United States. J. Medical Internet Res. 2020;22(6):e19782. doi: 10.2196/19782.

8. An L, Hawley S, Van Horn ML, et al. Development of a coronavirus social distance attitudes scale. Patient Educ Couns. 2020;104(6):1451-1459. doi: 10.1016/j.pec.2020.11.027.

9. Iwate Prefecture, Japan. Information on Confirmed Cases in Iwate. 2021. https://www.pref.iwate.jp/kurashikankyou/iryou/covid19/index.html (accessed November 19 2021).

10. The microCOVID Project, San Francisco. microCOVID Project. 2020. https://www.microcovid.org (accessed 27 November 2020).

For peer review only - http://bmjopen.bmj.com/site/about/guidelines.xhtml

11. Bavel JJV, Baicker K, Boggio PS, et al. Using social and behavioural science to support COVID-19 pandemic response. Nat Hum Behav 2020;4(5):460-71. doi: 10.1038/s41562-020-0884-z.

12. Chan HF, Skali A, Savage DA, et al. Risk attitudes and human mobility during the COVID-19 pandemic. Sci. Rep. 2020;10(1):19931. doi: 10.1038/s41598-020-76763-2

13. Pan Y, Darzi A, Kabiri A, et al. Quantifying human mobility behaviour changes during the COVID-19 outbreak in the United States. *Sci Rep* 2020;10(1):20742. doi: 10.1038/s41598-020-77751-2 [published Online First: 2020/11/28]

14. Lietz J, Westermann C, Nienhaus A, et al. The Occupational Risk of Influenza A (H1N1) Infection among Healthcare Personnel during the 2009 Pandemic: A Systematic Review and Meta-Analysis of Observational Studies. PLoS ONE 2016;11(8):e0162061. doi: 10.1371/journal.pone.0162061.

15. Marinaccio A, Boccuni F, Rondinone BM, et al. Occupational factors in the COVID-19 pandemic in Italy: compensation claims applications support establishing an occupational surveillance system. Occup Environ Med 2020;77(12):818-21. doi: 10.1136/oemed-2020-106844.

16. Nguyen LH, Drew DA, Graham MS, et al. Risk of COVID-19 among front-line health-care workers and the general community: a prospective cohort study. Lancet Public Health 2020;5(9):e475-e83. doi: 10.1016/s2468-2667(20)30164-x.

17. Mutambudzi M, Niedwiedz C, Macdonald EB, et al. Occupation and risk of severe COVID-19: prospective cohort study of 120 075 UK Biobank participants. Occup Environ Med 2020;0:1-8. doi: 10.1136/oemed-2020-106731.

For peer review only - http://bmjopen.bmj.com/site/about/guidelines.xhtml

18. Viner RM, Mytton OT, Bonell C, et al. Susceptibility to SARS-CoV-2 Infection Among Children and Adolescents Compared With Adults: A Systematic Review and Meta-analysis. JAMA Pediatr 2021;175(2):143-56. doi: 10.1001/jamapediatrics.2020.4573.

19. Yung CF, Kam K-q, Nadua KD, et al. Novel Coronavirus 2019 Transmission Risk in Educational Settings. Clin. Infect. Dis. 2020;72(6):1055-1058. doi: 10.1093/cid/ciaa794.

20. Kurita J, Sugawara T, Ohkusa Y. The second COVID-19 emergency status declaration for Japan: effects as of February 7, 2021. medRxiv 2021:2020.12.29.20248977. doi: 10.1101/2020.12.29.20248977

21. Centers for Disease Control and Prevention, USA. Information for Pediatric Healthcare Providers. 2020. https://www.cdc.gov/coronavirus/2019-ncov/hcp/pediatric-hcp.html (accessed 31 March 2021).

22. Vlachos J, Hertegård E, B. Svaleryd H. The effects of school closures on SARS-CoV-2 among parents and teachers. PNAS 2021;118(9):e2020834118. doi: 10.1073/pnas.2020834118.

23. Ministry of Health, Labour and Welfare, Japan. 22th meeting of the COVID-19 advisory board of Ministry of Health, Labour and Welfare. 2021. https://www.mhlw.go.jp/content/10900000/000759091.pdf (accessed 1 May 2021).

For peer review only

For peer review only - http://bmjopen.bmj.com/site/about/guidelines.xhtml

Figure Legends

Fig. 1. Flow chart of the selection of respondents.

Of 100,958 registered residents, 9,741 residents were selected for this study.

Fig. 2. Analytic models for behavioral risk trajectories.

For peer review only

For peer review only - http://bmjopen.bmj.com/site/about/guidelines.xhtml

Supplementary data

S1 Fig. Map of the study area.

Gray area represents Iwate, Japan.

S1 Text. Detailed calculation of microCOVID.

S1 Table. Weighting coefficient in items in each factor in activity and person risk.

S2 Table. Results of analysis using models for risk trajectories by multiple imputation methods (n=9,876).

S3 Table. Comparison of characteristics of the participants in the first survey and the characteristics of the whole population in the 2020 local census in Iwate.

S4 Table. Results of analysis using models for risk trajectories with survey weights (n=9,741)

S5 Table. Comparison of baseline characteristics of the non-participants and participants at the follow-up survey

For peer review only - http://bmjopen.bmj.com/site/about/guidelines.xhtml

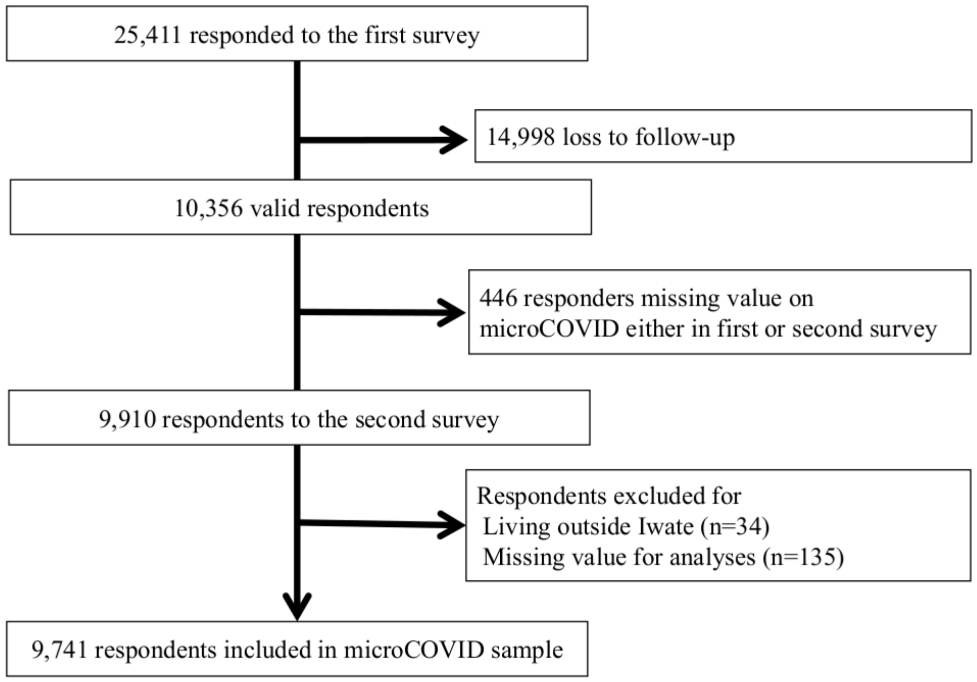

Fig. 1. Flow chart of the selection of respondents.

82x58mm (300 x 300 DPI)

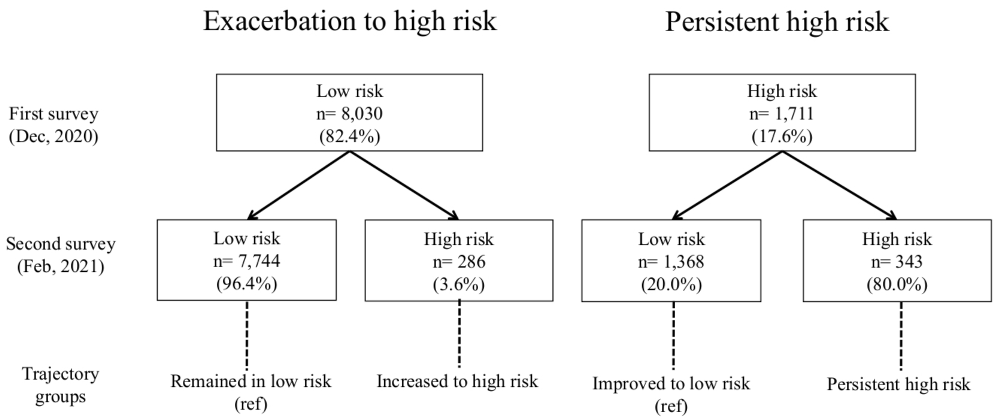

Fig. 2. Analytic models for behavioral risk trajectories.

112x50mm (300 x 300 DPI)

Supplementary data

S1 Text. Detailed calculation of microCOVID.

The microCOVID project is a calculator to measure and numerically quantify the risk of getting COVID-19 from an activity or relationship in participants' daily life based on available evidence. 1 microCOVID means a one-in-a-million chance of getting COVID. Microcovids are computed by using three major factors: activity risk, person risk, and number of people. We obtained the value by multiplying person interactions: microCOVID = Activity Risk x Number of people x Person Risk for one person.

Activity risk indicates the chance that the activity will transmit COVID from one to the other person who currently has COVID. In accordance with a previous study conducted by The Behavioral Insights Team in the UK [1], we modified some items in the questionnaire by asking respondents "did you meet anyone outside your household (or support bubble) in a risky situation in the past week?". A microCOVID value of zero was given to respondents who did not meet any person. Respondents who answered that they met someone went to the next questions asked about the details of the microCOVIDs value. Activity risk is a weighting based on coefficients how the interaction in the question is different from the reference value. Coefficients of risk are assigned to other types of interaction which differ from that reference value (if one person who has COVID, for 1 hour, indoors, unmasked, at 3 feet, there is a baseline 9% chance of transmission per hour) based on the following factors: duration of interaction, mask wearing (respondents and other persons), indoor/outdoor environment (indoor, outdoor, indoor with a HEPA filter, a train with air filtration, an airplane, a moving car with the windows rolled down, a space with one or more sides open to the outdoors), distance from each other, volume of conversation, and frequency (times a week). We calculated Activity Risk by multiplying 9%: Activity risk = coefficients x 9% (**S1 Table**).

For peer review only - http://bmjopen.bmj.com/site/about/guidelines.xhtml

With regard to the number of people, we asked respondents “how many people were there within a 5 meter radius of the scene?”.

Person risk represents the chance that the other person currently has COVID based on overall prevalence in the person’s area and recent behaviors of the person. Because the chance of a person having COVID depends not only on whether that person has any symptoms but also on the actions and choices of that person in the past 10 days, we used three different methods for estimating the probability of a person having COVID: a basic method, an intermediate method and an advanced method. The basic method is just an assumption that a person is “average” for their region. The probability of your friend having COVID is the probability of anyone in their geographic area having COVID. New infections in the past week were calculated as follows: new infections in the past week = reported cases x underreporting factor x delay factor. Based on that value, we estimated the person risk as follows: person risk (basic) = New infections in the past week / population in millions. The intermediate method is a quick adjustment for the risk a person being exposed at his/her workplace. The advance method involves adding up the risk of each individual activity that the person has done recently with consideration of three categories: socializing, errands, and work. In the intermediate and advanced methods, we calculated person risk by multiplying person risk (basic) by weighting coefficients in the following 12 items (**S1 Table**): “being an essential worker (working in health care, transportation or retailing)”, “living alone and only going to grocery stores for shopping”, “living in a closed pod of 4 people”, “living in a closed pod of 10 people”, “living in a closed pod of 20 people”, “having gone to a bar in the past 10 days”, “going to a bar frequently”, “having been a patient with COVID-19”, “you and other persons living with you only going to grocery stores for shopping”, “other persons living with

For peer review only - http://bmjopen.bmj.com/site/about/guidelines.xhtml

48 you not being essential workers", "not have any interaction with others and other persons living with you who are essential workers", and "not applicable for those items".

For peer review only

For peer review only - http://bmjopen.bmj.com/site/about/guidelines.xhtml

S1 Table. Weighting coefficient in items in each factor in activity and person risk.

Risk	Factors	Items	Coefficient
**Activity Risk**	**Respondents' mask**	No mask or inappropriate usage	1
		Polyester or polyurethane mask	0.5
		Cloth or bandanna	0.25
		Surgical mask, non-woven mask	0.25
		N95 mask	0.1
	**Other person's mask**	No mask or inappropriate usage	1
		Polyester or polyurethane mask	1
		Cloth or bandanna	1
		Surgical mask, non-woven mask	0.5
		N95 mask	0.1
	**Indoor/outdoor environment**	Indoor without ventilation	1
		Outdoor	0.05
		Indoor with a HEPA filter	0.25
		A train with air filtration	0.25
		An airplane	0.17
		A moving car with the windows rolled down	0.25
		A space with one or more sides open to the outdoors	0.25
	**Distance from each other**	< 0.3 meters apart	2

For peer review only

		About 1 meter apart	1
		2+ meters apart	0.5
		3+ meters apart	0.25
	**Volume of conversation**	Not talking	0.2
		Normal conversation	1
		Loud talking (shouting, talking over music, singing)	5
**Person Risk**	**Intermediate and Advanced Method**	Being an essential workers	2
		Living alone and only going to grocery stores for shopping	0.011
		Living in a closed pod of 4 people	0.021
		Living in a closed pod of 10 people	0.041
		Living in a closed pod of 20 people	0.075
		Having gone to a bar in the past 10 days	14.5
		Going to a bar frequently	0.9
		Having been a patient with COVID-19	1000000
		You and other persons living with you only going to grocery stores for shopping	0.014
		Other persons living with you not being essential workers	0.5
		Not have any interaction with others and other persons living with you who are essential workers	0.61
		Not applicable for those items	1

For peer review only - http://bmjopen.bmj.com/site/about/guidelines.xhtml

S2 Table. Results of analysis using models for risk trajectories by multiple imputation methods (n=9,876).

		Model for transition to high risk (n=8,113)		Model for high risk persistence (n=1,763)	
		OR (95% CI)	P-value	OR (95% CI)	P-value
**Age groups**	**Young (ref: elderly)**	1.96 (1.19 - 3.24)	0.008	1.59 (0.90 - 2.81)	0.111
	**Middle age**	1.51 (0.93 - 2.44)	0.092	1.49 (0.86 - 2.57)	0.157
**Sex**	**Women (ref: men)**	1.05 (0.80 - 1.37)	0.733	1.25 (0.91 - 1.71)	0.17
**Area**	**Inland areas (ref: coastal and mountainous areas)**	0.99 (0.75 - 1.32)	0.958	0.83 (0.62 - 1.11)	0.207
**Occupation**	**Health care workers (ref: government workers)**	2.66 (1.76 - 4.01)	<0.001	1.78 (1.17 - 2.73)	0.008
	**Service**	1.18 (0.78 - 1.78)	0.437	1.51 (0.97 - 2.35)	0.07
	**Schools**	1.17 (0.69 - 1.98)	0.569	2.62 (1.59 - 4.34)	<0.001
	**Others**	0.56 (0.35 - 0.88)	0.013	1.23 (0.72 - 2.10)	0.451
**Rate of decrease in eating out compared with that in the past year**	**Decreased by 50-70% (ref: decreased by 80% or more)**	1.22 (0.91 - 1.64)	0.183	1.03 (0.76 - 1.40)	0.83
	**Decreased by 40% or less**	1.02 (0.75 - 1.39)	0.888	1.21 (0.89 - 1.64)	0.22
**Homecoming in the New Year's holiday season**	**Yes (ref: no)**	1.04 (0.75 - 1.44)	0.809	1.17 (0.85 - 1.59)	0.334
**The first shrine visit of the year**	**Do not visit every year (ref: not visited on this year for prevention)**	1.08 (0.76 - 1.55)	0.664	1.81 (1.23 - 2.66)	0.003
	**Visited**	1.03 (0.78 - 1.35)	0.853	1.12 (0.85 - 1.49)	0.414
**Measures in the second state of emergency**	**Lower (ref: higher)**	1.59 (1.04 - 2.44)	0.032	1.28 (0.85 - 1.94)	0.244
	**Same**	0.90 (0.65 - 1.23)	0.487	0.77 (0.57 - 1.04)	0.085

Abbreviations: CI, confidence interval; OR, odds ratio

For peer review only - http://bmjopen.bmj.com/site/about/guidelines.xhtml

55 **S3 Table. Comparison of characteristics of the participants in the first survey and the**

56 **characteristics of the whole population in the 2020 local census in Iwate.**

57

		**2020 local census**	**All analytic sample in the first survey**
		**n (%)**	**n (%)**
**Sex**	**Men**	585077 (48.3)	2692 (27.6)
	**Women**	627124 (51.7)	7049 (72.4)
**Age classes**	**Young**	396943 (32.7)	2765 (28.4)
	**Middle age**	315660 (26.0)	5802 (59.6)
	**Elderly**	492455 (40.6)	1174 (12.1)
	**Unknown**	7143 (0.6)	Not applicable
**Area**	**Inland areas**	928199 (76.6)	7859 (80.7)
	**Coastal and mountainous areas**	284002 (23.4)	1882 (19.3)

58

For peer review only

For peer review only - http://bmjopen.bmj.com/site/about/guidelines.xhtml

S4 Table. Results of analysis using models for risk trajectories with survey weights (n=9,741)

		Model for transition to high risk (n=8030)		Model for high risk persistence (n=1711)	
		OR (95% CI)	P-value	OR (95% CI)	P-value
Age groups	Young (ref: elderly)	2.56 (1.51 - 4.33)	<0.001	1.37 (0.71 - 2.65)	0.342
	Middle age	1.78 (0.99 - 3.19)	0.052	1.47 (0.72 - 2.98)	0.288
Sex	Women (ref: men)	1.09 (0.76 - 1.56)	0.652	1.31 (0.88 - 1.95)	0.19
Area	Inland areas (ref: coastal and mountainous areas)	1.75 (1.14 - 2.70)	0.011	1.00 (0.65 - 1.54)	0.987
Occupation	Health care workers (ref: government workers)	1.74 (1.01 - 2.98)	0.045	1.42 (0.75 - 2.66)	0.28
	Service	0.99 (0.60 - 1.63)	0.958	1.08 (0.57 - 2.08)	0.808
	Schools	0.53 (0.24 - 1.15)	0.107	1.60 (0.79 - 3.25)	0.19
	Others	0.65 (0.37 - 1.12)	0.121	0.54 (0.22 - 1.30)	0.168
Rate of decrease in eating out compared with that in the past year	Decreased by 50-70% (ref: decreased by 80% or more)	1.17 (0.77 - 1.78)	0.467	1.40 (0.88 - 2.23)	0.153
	Decreased by 40% or less	1.38 (0.92 - 2.07)	0.122	1.44 (0.89 - 2.34)	0.139
Homecoming in the New Year's holiday season	Yes (ref: no)	0.86 (0.56 - 1.33)	0.501	1.05 (0.67 - 1.63)	0.847
The first shrine visit of the year	Do not visit every year (ref: not visited on this year for prevention)	0.99 (0.59 - 1.67)	0.98	2.72 (1.48 - 5.01)	0.001
	Visited	1.03 (0.72 - 1.49)	0.868	2.05 (1.26 - 3.34)	0.004
Measures in the second state of emergency	Lower (ref: higher)	1.44 (0.85 - 2.44)	0.178	1.33 (0.73 - 2.43)	0.358
	Same	0.73 (0.48 - 1.09)	0.127	0.66 (0.41 - 1.05)	0.081

Abbreviations: CI, confidence interval; OR, odds ratio

For peer review only - http://bmjopen.bmj.com/site/about/guidelines.xhtml

S5 Table. Comparison of baseline characteristics of the non-participants and participants at the follow-up survey

		Missing	Non-participants (n=15055)	Study participants (n=10356)	*P* value
		n (%)	n (%)	n (%)	
**Age groups**	**Young**	0 (0.0)	4638 (30.8)	2950 (28.5)	<0.001
	**Middle age**		8544 (56.8)	6153 (59.4)	
	**Elderly**		1873 (12.4)	1253 (12.1)	
**Sex**	**Women**	117 (0.0)	10215 (68.2)	7420 (72.0)	<0.001
**Area**	**Inland areas**	0 (0.0)	12322 (81.8)	8311 (80.3)	<0.001
**Occupation**	**Health care workers**	0 (0.0)	3323 (22.1)	2117 (20.4)	<0.001
	**Service**		4992 (33.2)	3048 (29.4)	
	**Schools**		1158 (7.7)	859 (8.3)	
	**Others**		4202 (27.9)	3112 (30.1)	
	**Government workers**		1380 (9.2)	1220 (11.8)	

P values were calculated using the chi-squared test for categorical variables.

For peer review only - http://bmjopen.bmj.com/site/about/guidelines.xhtml

References

1. Yihan X, Mark E, Tania L, Lev T, Abigail M, Hugo H. A small number of people account for a large amount of coronavirus risk. In: team Tbi, ed. United Kingdom: The behavioural insights team, 2020.

For peer review only

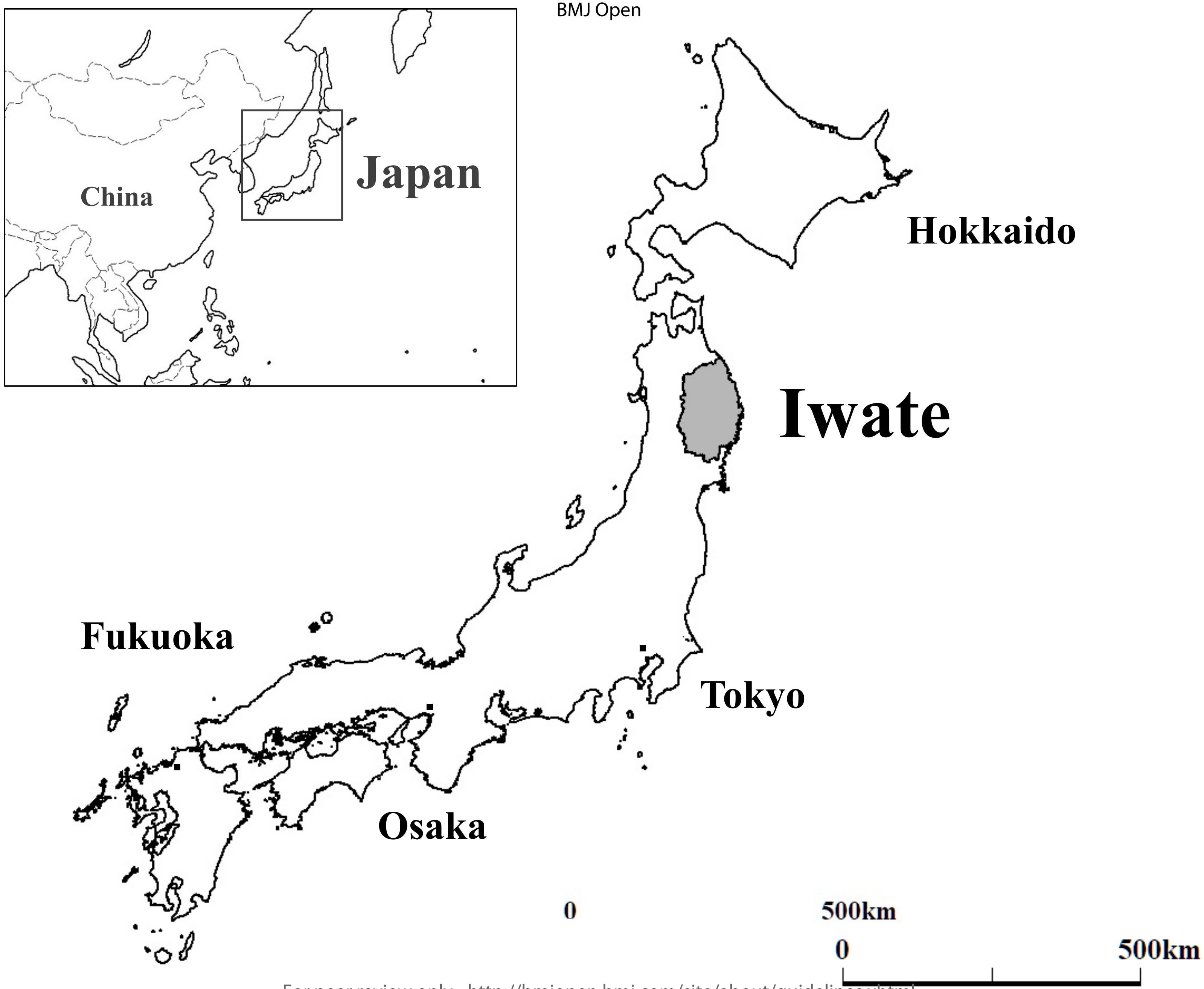
China
Japan
Hokkaido
Iwate
Fukuoka
Tokyo
Osaka
0
500km
0
500km

STROBE 2007 (v4) checklist of items to be included in reports of observational studies in epidemiology*
Checklist for cohort, case-control, and cross-sectional studies (combined)

Section/Topic	Item #	Recommendation	Reported on page #
**Title and abstract**	1	(*a*) Indicate the study's design with a commonly used term in the title or the abstract	Page 1
		(*b*) Provide in the abstract an informative and balanced summary of what was done and what was found	Page 2-3
**Introduction**			
Background/rationale	2	Explain the scientific background and rationale for the investigation being reported	Page 4-5
Objectives	3	State specific objectives, including any pre-specified hypotheses	Page 5
**Methods**			
Study design	4	Present key elements of study design early in the paper	Page 5-6
Setting	5	Describe the setting, locations, and relevant dates, including periods of recruitment, exposure, follow-up, and data collection	Page 5-6, Fig .1-2, Supplementary S1 Fig
Participants	6	(*a*) *Cohort study*—Give the eligibility criteria, and the sources and methods of selection of participants. Describe methods of follow-up<br>*Case-control study*—Give the eligibility criteria, and the sources and methods of case ascertainment and control selection. Give the rationale for the choice of cases and controls<br>*Cross-sectional study*—Give the eligibility criteria, and the sources and methods of selection of participants	Page 5-6
		(*b*) *Cohort study*—For matched studies, give matching criteria and number of exposed and unexposed<br>*Case-control study*—For matched studies, give matching criteria and the number of controls per case	Page 5-6
Variables	7	Clearly define all outcomes, exposures, predictors, potential confounders, and effect modifiers. Give diagnostic criteria, if applicable	Page 6-8
Data sources/ measurement	8*	For each variable of interest, give sources of data and details of methods of assessment (measurement). Describe comparability of assessment methods if there is more than one group	Page 6-8
Bias	9	Describe any efforts to address potential sources of bias	Page 12
Study size	10	Explain how the study size was arrived at	Page 5-6
Quantitative variables	11	Explain how quantitative variables were handled in the analyses. If applicable, describe which groupings were chosen and why	Page 6-7, Supplementary S1 Text
Statistical methods	12	(*a*) Describe all statistical methods, including those used to control for confounding	Page 8
		(*b*) Describe any methods used to examine subgroups and interactions	Page 6-8
		(*c*) Explain how missing data were addressed	Page 8

For peer review only

		(*d*) *Cohort study*—If applicable, explain how loss to follow-up was addressed<br>*Case-control study*—If applicable, explain how matching of cases and controls was addressed<br>*Cross-sectional study*—If applicable, describe analytical methods taking account of sampling strategy	Page 8
		(*e*) Describe any sensitivity analyses	Page 8
**Results**			
Participants	13*	(a) Report numbers of individuals at each stage of study—eg numbers potentially eligible, examined for eligibility, confirmed eligible, included in the study, completing follow-up, and analysed	Page 6
		(b) Give reasons for non-participation at each stage	Page 6
		(c) Consider use of a flow diagram	Page 6, Fig. 1
Descriptive data	14*	(a) Give characteristics of study participants (eg demographic, clinical, social) and information on exposures and potential confounders	Page 9, Table. 1
		(b) Indicate number of participants with missing data for each variable of interest	Fig. 1
		(c) *Cohort study*—Summarise follow-up time (eg, average and total amount)	Page 5-6, Fig. 2
Outcome data	15*	*Cohort study*—Report numbers of outcome events or summary measures over time	Page 6-7, Fig. 2
		Case-control study—Report numbers in each exposure category, or summary measures of exposure	
		Cross-sectional study—Report numbers of outcome events or summary measures	
Main results	16	(*a*) Give unadjusted estimates and, if applicable, confounder-adjusted estimates and their precision (eg, 95% confidence interval). Make clear which confounders were adjusted for and why they were included	Page 12-13, Table 2
		(*b*) Report category boundaries when continuous variables were categorized	Page 6-7, Supplementary S1 Text
		(*c*) If relevant, consider translating estimates of relative risk into absolute risk for a meaningful time period	
Other analyses	17	Report other analyses done—eg analyses of subgroups and interactions, and sensitivity analyses	Page 14, S2 Table
**Discussion**			
Key results	18	Summarise key results with reference to study objectives	Page 14
Limitations	19	Discuss limitations of the study, taking into account sources of potential bias or imprecision. Discuss both direction and magnitude of any potential bias	Page 17
Interpretation	20	Give a cautious overall interpretation of results considering objectives, limitations, multiplicity of analyses, results from similar studies, and other relevant evidence	Page 14-16
Generalisability	21	Discuss the generalisability (external validity) of the study results	Page 17
**Other information**			
Funding	22	Give the source of funding and the role of the funders for the present study and, if applicable, for the original study on which the present article is based	Page 18

For peer review only

*Give information separately for cases and controls in case-control studies and, if applicable, for exposed and unexposed groups in cohort and cross-sectional studies.

Note: An Explanation and Elaboration article discusses each checklist item and gives methodological background and published examples of transparent reporting. The STROBE checklist is best used in conjunction with this article (freely available on the Web sites of PLoS Medicine at http://www.plosmedicine.org/, Annals of Internal Medicine at http://www.annals.org/, and Epidemiology at http://www.epidem.com/). Information on the STROBE Initiative is available at www.strobe-statement.org.

For peer review only
